# Structural and functional analysis of Bacillus cereus spore cortex lytic enzymes and YlaJ/YhcN lipoproteins

**DOI:** 10.1099/mic.0.001591

**Published:** 2025-08-07

**Authors:** Amin Mustafa, Bahja Al Riyami, Giannina Ow-Young-Villarreal, Rebecca Caldbeck, David Chan Hian Pin, Joshua Yarrow, Graham Christie

**Affiliations:** 1 Department of Chemical Engineering and Biotechnology, University of Cambridge, Cambridge, UK

**Keywords:** *Bacillus cereus*, cortex lytic enzymes (CLEs), germination, lipoproteins, spores

## Abstract

Resumption of the planktonic phase of the *Bacillus cereus* cell cycle necessitates degradation of certain morphological structures and physiological features that confer metabolic dormancy and multi-factorial resistance properties to the spore form of the bacterium. Depolymerization of the peptidoglycan cortex, which is crucial to maintenance of spore dormancy, constitutes a major germination event and is conducted by a complement of spore cortex lytic enzymes that are active only during spore germination. This work reports on the structure and function of the major cortex lytic enzymes in *B. cereus* spores, revealing insight to their location, individual contributions to germination when triggered by different routes and regions of the SleB protein that are important for mediating interactions with its peptidoglycan substrate. The effect of null mutations to lipoproteins of the YlaJ/YhcN family on spore properties is also characterized, revealing parallels with prior observations concerning YlaJ’s influence on SleB activity during germination. Finally, a structural model of a putative SleB-YpeB-YlaJ complex is presented. The model, which was subject to an initial validation by evolutionary covariance analysis and site-directed mutagenesis, reveals how the SleB protein might be held in an inactive state courtesy of its interactions with YpeB and YlaJ during spore dormancy, potentially shedding light on a long-standing puzzle in spore germination.

**Impact Statement**

*Bacillus cereus* is a major causative agent of food-borne toxigenesis. The bacterium’s ability to form heat-resistant spores is a significant contributing factor in this regard since the spore form of the organism can often survive conditions encountered during food processing or cooking. However, only the vegetative form of the bacterium is responsible for producing toxins; hence, the spore has to germinate in order to present a threat. A crucial step in germination concerns degradation of the spore’s thick layer of peptidoglycan or cortex. This study reveals fresh insight into the enzymes that are responsible for this process, including a structural model that demonstrates how one of the major cortex lysins is held in an inactive state during spore dormancy. Knowledge in this regard has important practical implications since it may lead to the development of improved approaches for the eradication of spores from food.

## Data Availability

Research data associated with this manuscript have been deposited in the University of Cambridge Apollo repository at https://doi.org/10.17863/CAM.120070.

## Introduction

Members of the *Bacillus* genus of bacteria, a taxonomic division of the order Bacillales, are characterized by their ability to sporulate upon nutrient starvation. The resultant spores are among the most resilient cell types found in nature, with several unique morphological and physiological adaptations that permit their survival in the environment until they are stimulated to germinate and resume planktonic or sessile growth [1]. The initial stages of the germination process are mediated by various receptor and ion channel complexes, which result in significant efflux of ions and analytes from – and partial hydration of – the spore core [2,4]. Soon after, the thick layer of structurally distinct peptidoglycan that is critical to the maintenance of spore dormancy, and which is referred to as the cortex, is degraded by specialized peptidoglycan lysins. The activity of these cortex lytic enzymes (CLEs) permits complete hydration of the core and ultimately the resumption of metabolic activity and vegetative outgrowth. Spores of most species are equipped typically with many different CLEs, but only two, SleB and CwlJ, are capable of initiating cortex hydrolysis [2]. CLEs have been characterized by mutagenesis in spores of several species, including *Bacillus subtilis*, *Bacillus anthracis*, *Bacillus megaterium* and *Bacillus licheniformis*, but not in *Bacillus cereus* [5,8]. This seems something of an oversight given that the latter is a significant human pathogen responsible for tens of thousands of food-related gastrointestinal illnesses per annum in the USA alone, and the CLEs are potential targets for spore intervention measures. Hence, we decided to address this knowledge gap in the course of this work by investigating the effect of various CLE and associated lipoprotein null mutations on *B. cereus* spore germination and viability, while conducting more precise work on the role of the N-terminal peptidoglycan binding domain of SleB. We also present a structural model of a SleB-YpeB-YlaJ complex, which offers clues to how SleB may be held in check during spore dormancy.

## Methods

### Bacterial strains and spore preparation

*B. cereus* strains employed in this study were isogenic with the ATCC 10876 or ATCC 14579 strains (Tables 1 and S1, available in the online Supplementary Material). *B. cereus* strains were routinely cultured in Lysogeny Broth (LB) medium at 30 °C, supplemented with antibiotics where appropriate (Table 1). Competent *Escherichia coli* DH5α cells were used for cloning procedures and for the propagation of plasmids. *B. cereus* spores were prepared by nutrient exhaustion in supplemented nutrient broth (SNB) medium, comprising the following (per litre): Difco nutrient broth, 8.0 g; glucose, 1.0 g; KCl, 1.0 g; MgSO_4_.7H_2_O, 246 mg; CaCl_2_.2H_2_O, 147 mg; MnCl_2_. 4H_2_O, 4 mg; and FeSO_4_.7H_2_O, 0.3 mg. The pH was adjusted to 7.2 prior to autoclaving. Efficient sporulation was achieved in 2 l baffled flasks containing 200 ml medium that was inoculated with 1 ml of cells from an overnight culture which was then subject to orbital agitation at 30 °C for 48 h. Spores were purified from cellular debris by several cycles of centrifugation and resuspension in ice-cold deionized water until phase-bright spores were observed by microscopy to constitute >99% of the suspension. Purified spores were stored in water at 4 °C, an OD at 600 nm of ~50.

**Table 1.** Isogenic derivative strains of *B. cereus* 10876 used in this work and viability of their spores*

Strain	Genotype	Viability (%)†	Source (reference)
10876	Wild type	100	Anne Moir
Null mutant strains			
AM029	*ΔsleB*	29	This work
AM033	*ΔcwlJ1*	100	This work
AM034	*ΔcwlJ2*	97	This work
AM035	*ΔsleB ΔcwlJ1*	0.002	This work
AM036	*ΔsleB ΔcwlJ2*	38	This work
AM023	*ΔcwlJ1 ΔcwlJ2*	41	This work
AM027	*ΔsleB ΔcwlJ1 ΔcwlJ2*	0.0002	This work
AM020	*ΔylaJ*	38	This work
AM021	*ΔyhcN*	81	This work
AM022	*ΔylaJ ΔyhcN*	40	This work
AM024	*ΔcwlJ1 ΔcwlJ2 ΔylaJ*	39	This work
AM025	*ΔcwlJ1 ΔcwlJ2 ΔyhcN*	44	This work
AM026	*ΔcwlJ1 ΔcwlJ2 ΔylaJ ΔyhcN*	18	This work
AM039	*ΔgerE*	–	This work
Fluorescence reporter strains†			
AM078	pHT-P*_sleB_-sleB^N^-tdTomato*	–	This work
AM076	pHT-P*_cwlJ1_-cwlJ1-gfp*	–	This work
AM077	pHT-P*_cwlJ2_-cwlJ2-gfp*	–	This work
*ΔgerE* SleB^N^-tdTomato fusions†			
AM159	*ΔgerE* pHT-P*_sleB_-sleB^N^-tdTomato*	–	This work
AM160	*ΔgerE* pHT-P*_sleB_-sleB^N^* ^F33A^*-tdTomato*	–	This work
AM161	*ΔgerE* pHT-P*_sleB_-sleB^N^* ^V37A^*-tdTomato*	–	This work
AM168	*ΔgerE* pHT-P*_sleB_-sleB^N^* ^D65A^*-tdTomato*	–	This work
AM167	*ΔgerE* pHT-P*_sleB_-sleB^N^* ^T72A T95A^*-tdTomato*	–	This work
AM164	*ΔgerE* pHT-P*_sleB_-sleB^N^* ^D88A^*-tdTomato*	–	This work
AM165	*ΔgerE* pHT-P*_sleB_-sleB^N^* ^L90A^*-tdTomato*	–	This work
AM166	*ΔgerE* pHT-P*_sleB_-sleB^N^* ^T95A^*-tdTomato*	–	This work
AM162	*ΔgerE* pHT-P*_sleB_-sleB^N^* ^K96A^*-tdTomato*	–	This work
AM163	*ΔgerE* pHT-P*_sleB_-sleB^N^* ^V100A^*-tdTomato*	–	This work
*ΔsleB ΔcwlJ1 ΔcwlJ2* (*CLE3*) SleB variant strains†			
GOV01	*CLE3 pHT315*	–	This work
GOV02	*CLE3* pHT-P*_sleB_-sleB-ypeB-ylaJ*	–	This work
GOV03	*CLE3* pHT-P*_sleB_-sleB^D65A^-ypeB-ylaJ*	–	This work
GOV04	*CLE3* pHT-P*_sleB_-sleB^D88A^-ypeB-ylaJ*	–	This work
GOV05	*CLE3* pHT-P*_sleB_-sleB^D65A D88a^-ypeB-ylaJ*	–	This work
GOV06	*CLE3* pHT-P*_sleB_-sleB^K214A^-ypeB-ylaJ*	–	This work
GOV07	*CLE3* pHT-P*_sleB_-sleB^D218A^-ypeB-ylaJ*	–	This work
GOV08	*CLE3* pHT-P*_sleB_-sleB^K250A^-ypeB-ylaJ*	–	This work
GOV09	*CLE3* pHT-P*_sleB_-sleB-ypeB^D322A^-ylaJ*	–	This work
GOV10	*CLE3* pHT-P*_sleB_-sleB-ypeB^K346A^-ylaJ*	–	This work
GOV11	*CLE3* pHT-P*_sleB_-sleB-ypeB^D270A^-ylaJ*	–	This work
GOV12	*CLE3* pHT-P*_sleB_-sleB^D218A^-ypeB^D322A^-ylaJ*	–	This work
GOV13	*CLE3* pHT-P*_sleB_-sleB-ypeB^D270A D322A^-ylaJ*	–	This work
GOV14	*CLE3* pHT-P*_sleB_-sleB^D218A^-ypeB^D270A D322A^-ylaJ*	–	This work

*Spores of various strains were prepared and purified, and their relative viability was determined as described in the Methods section. All values shown are averages of results with two independent spore preparations and are ±25% of the value shown.

†These strains are resistant to erythromycin (1 µg ml−1) and lincomycin (25 µg ml−1).

### Molecular biology procedures

Genes of interest were deleted using the markerless allelic exchange technique described previously [9]. Essentially, PCR was used to produce 500 bp amplicons upstream of the start codon and 500 bp downstream of the stop codon of the ORF, respectively. Oligonucleotide primers included ~25 bp of overlapping sequence between the 3′ end of the upstream amplicon and the 5′ end of the downstream amplicon (primer sequences are shown in Tables S8–S11). Additionally, the 5′ end of the upstream amplicon contained 25 bp of overlapping sequence with the 5′ end of EcoRI and BamHI-digested pMAD vector [10], as did the 3′ end of the downstream amplicon with the 3′ end of the linearized vector. The fragments were assembled using the ligase-free Klenow Assembly Method (https://www.researchgate.net/publication/320552155_Klenow_assembly_method_seamless_cloning) before introducing to chemically competent *E. coli*. Carbenicillin-resistant colonies were screened by PCR to identify clones with the intended construct prior to plasmid purification and sequencing. Verified pMAD plasmids were demethylated by passaging through *dam-/dcm- E. coli* (New England Biolabs, UK) before introducing to *B. cereus* by electroporation and selecting for transformants on LB plates supplemented with 1 µg ml^−1^ erythromycin, 5 µg ml^−1^ lincomycin and 90 µg ml^−1^ X-gal. Efficient electroporation was achieved using a Gene Pulser instrument (Bio-Rad) operating at 200 Ω, 2 kV and 25 µF with cuvettes that contained 500 ng of plasmid DNA plus 50 µl of thawed electrocompetent cells. Representative blue colonies were then used to inoculate 2 ml LB medium containing the same supplements before culturing at 37 °C for 72 h, sub-culturing at 24 h intervals. Electrocompetent versions of these single-crossover cells were then transformed to tetracycline resistance with the I-SceI-encoding pBKJ223 plasmid, which promotes an efficient second recombination event at the pMAD integration site. Representative transformants were cultured at 37 °C in 2 ml LB medium containing 10 µg ml^−1^ tetracycline for three consecutive days, again subculturing at 24 h intervals, before plating dilute aliquots on solid LB medium supplemented with tetracycline and X-gal. Candidate (white) colonies that had excised the integrated pMAD plasmid, leaving behind only the start and stop codons of the truncated gene, were validated by PCR and sequencing and then passaged on antibiotic-free LB medium until the pBKJ223 plasmid had been excised from mutant strains. The same procedure was repeated where necessary to construct strains with multiple gene deletions. Complementation assays were conducted *in trans*, using the low copy number pHT315 plasmid [11] as the backbone for cloned inserts of interest. Functional analysis of variant SleB proteins, for example, was achieved by cloning the entire PCR-amplified *sleB-ypeb-ylaJ* operon, including the upstream promoter sequence, into linearized pHT315, using the Klenow Assembly Method, and then introducing the sequence-verified plasmid to the *B. cereus* 10876 *sleB ypeB* null strain via electroporation. A QuikChange Lightning Site-Directed Mutagenesis kit (Agilent Technologies, Stockport, UK) was used to modify codons of interest in the *sleB* ORF using the aforementioned pHT-*sleB* operon plasmid as a template.

Fluorescence reporter strains were constructed by using PCR to prepare DNA amplicons encoding ORFs, for example, *B. cereus* ATCC 14579 SleB^N^ and its upstream promoter sequence and the tdTomato fluorescent protein. The amplicons were subsequently purified and cloned into linearized pHT315 using the Klenow Assembly Method, creating an ORF that encoded an in-frame SleB^N^-tdTomato protein under control of the native promoter sequence. The *sleB^N^-tdTomato* ORF additionally encoded a flexible linker sequence (GGGEAAAKGGG) between the SleB^N^ and tdTomato domains. The pHT315-*sleB^N^-tdTomato* plasmid was used subsequently as template DNA for PCR reactions designed to create a number of variant SleB^N^-tdTomato proteins with defined alanine substitutions (Table 1). Correct construction of the various pHT315-derived plasmids was verified by DNA sequencing prior to demethylation and introduction by electroporation to *B. cereus* 10876 Δ*gerE* cells. The *gerE* gene (GenBank accession number EEK48648) was deleted using the markerless deletion approach described above. The various strains were then induced to sporulate by establishing 200 ml cultures in SNB medium supplemented with 1 µg ml^−1^ erythromycin at 30 °C, 225 r.p.m., for 48 h. Samples were withdrawn at various time points and subject to fluorescence microscopy (described below) with a view to examining localization of SleB^N^-tdTomato and variant proteins during sporulation. Constructs containing *gfp* or *tdTomato* fusions to *cwlJ1*, *cwlJ2*, *ylaJ* and *yhcN*, each under the control of their native promoter sequences, were prepared and examined using similar procedures. NCBI accession identifiers for proteins examined in this work are detailed in Table S2.

### Recombinant SleB^N^ expression, purification and analysis

Recombinant SleB^N^ with an N-terminal His_6_ tag was produced by introducing a PCR amplicon spanning residues A32–T103 into the pET28a(+) vector via Klenow Assembly. Amino acid substitutions (D65A and D88A) were introduced singly and combined via PCR and Klenow Assembly. Protein expression was conducted in *E. coli* BL21(DE3) cells at 10 °C for 5 days after inducing with 1 mM IPTG. Protein purification from cell lysates was achieved via sequential Ni-NTA affinity, ion-exchange and size exclusion chromatography steps using ÄKTA Pure instrumentation and with purity assessed by SDS-PAGE. Circular dichroism spectra were obtained using a Jasco J-1500 CD instrument operating at room temperature. Protein samples were presented in 20 mM NaPO_4_, pH 7.0, at a concentration of 2.3 µM. All measurements were taken in a quartz cuvette with a path length of 10 mm. Spectra ranging from 190 to 250 nm were collected with a bandwidth of 1 nm, scanning speed of 50 nm min^−1^ and data pitch of 0.1 nm and averaged across three accumulations. Spectra were smoothed via the Means-Movement method with a convolution width of 15.

### Microscopy

Samples for analysis were pipetted (3 µl) onto poly-l-lysine-coated microscope slides before sealing with glass coverslips. Samples were imaged on an Olympus BX53 microscope fitted with phase-contrast optics and a 100×1.30 numerical aperture oil objective lens. Fluorescence analysis was achieved via illumination from a mercury lamp and appropriate filters for GFP and red fluorescence. Images were captured with a Retiga-2000R charge-coupled-device camera, giving a pixel width of 74 nm on the specimen, and 12-bit grey levels. Images were recorded as 1,600 by 1,200 pixel Tiffs. The quantitative fluorescence ellipsoid localization microscopy (ELM) technique was used to measure the location of fluorescent fusion proteins in mature spores and in sporulating cells [12]. Time-lapse microscopy of spore germination was achieved using a standard agar pad approach, i.e. 2 µl of heat-shocked and cooled spore sample, resuspended in germinant containing buffer, was transferred rapidly to an agarose pad prepared from ~100 µl 2 wt% molten agarose, before covering with a glass cover slip and sealing with nail varnish prior to mounting on the microscope stage. ImageJ software was used to capture phase-contrast images at a sampling frequency of one frame per minute, typically for up to 3 h at room temperature. Micrographs were processed using the Image Stabiliser plugin and then subjected to SporeTracker analysis for the quantification of kinetic parameters associated with the germination of individual spores as described previously [13].

### Spore germination

Spore germination was monitored by measuring the absorbance at 600 nm (A600) of 200 µl spore suspensions in 96-well plates using a Tecan Infinite M200 plate reader (Tecan, UK). Spores were heat-shocked at 75 °C for 30 min and then cooled on ice before adding to 10 m Tris-HCl, pH 7.4, containing 10 mM l-alanine and 1 mM inosine at a concentration of ~10^8^ spores per millilitre. Plates were sealed with transparent film and germination monitored for 90–120 min at 37 °C with 1 min interval absorbance measurement followed by 10 s of orbital shaking to prevent spores from settling at the bottom of the wells. Presented data are from single experiments, which are representative of multiple analyses conducted with at least two independent batches of spores. Germination experiments conducted with non-physiological germinants were conducted by resuspending non-heat-shocked spores in 10 mM Tris-HCl, pH 7.4, supplemented with 50 mM CaDPA (at 30 °C) or 1 mM dodecylamine (at 40 °C). Changes in absorbance of the spore suspensions were monitored as described above. Spore colony-forming ability was assessed by plating serially diluted spore suspensions on LB agar plates, followed by colony enumeration after 24 h of incubation at 30 °C.

### Protein complex modelling and evolutionary coupling analysis

The original SleB^M^ (i.e. the mature protein comprising residues 33–259)+YpeB dimer-of-dimers model was predicted using AlphaFold2–Multimer [14,15]. The SleB+YpeB+YlaJ^17-198^ (17C: S-palmitoyl-l-cysteine) dimer-of-trimers model was predicted using AlphaFold 3 [16], as well as membrane approximation models which were produced by inserting as many palmitic acid (PLM ligand) molecules as possible within the token limit after setting up the protein chains (~150–200). Some alternative stoichiometries were tried but led to nonsensical and/or low-confidence results (data not shown). Evolutionary couplings for the SleB+YpeB multimer were calculated using the EVcouplings V1 server (https://v1.evcouplings.org) using default settings and a bitscore threshold of 0.3. Evolutionary couplings for the SleB+YlaJ and YpeB+YlaJ multimers were calculated on the Cambridge HPC Cluster, CSD3, using the equivalent EVCouplings script with a bitscore threshold of 0.7 and otherwise default settings. All protein input sequences were from the *B. cereus* ATCC 10876 strain. EVcouplings config files are provided in the Supplementary Material. A custom plotting Jupyter Notebook was used to visualize the ‘long-range couplings’ output from EVcouplings with multiple types of 3D contact (intra-chain, intra-protomer heteromer, inter-protomer homomer and inter-protomer heteromer), where the aim was to maximize visual similarity with the off-the-shelf visualizations provided by the EVcouplings package. For each pair of residues in the 3D models, distances between all pairs of atoms were calculated, and a contact was marked if any of them were within 5 Å. These plots also included an interactive tooltip which was used to easily identify couplings of interest; to do this, we started by producing multiple visualizations which thresholded the coupling probability at 0.99, 0.8 and 0.6. After inspecting these, additional plots were produced as required to manually locate couplings that were well balanced between high confidence and biochemical salience (as per the guidance in the EVcouplings literature which suggests that some lower-confidence couplings may be considered in the light of prior knowledge). For the SleB+YpeB model, a selection of couplings was used to construct a 3D visualization with sticks representing the couplings similar to those produced by the EVcouplings package but with distinct colours for each pair of protein chains. The most convincing couplings were inspected in the 3D model using PyMOL and were shortlisted for site-directed mutagenesis. The plotting code, relevant 3D models and EVcouplings output CSV files can be accessed at the University of Cambridge Apollo repository (https://doi.org/10.17863/CAM.120070).

## Results

### Characterization of *B. cereus* CLE null mutant spores

The *B. cereus* 10876 genome, in common with other members of the wider *B. cereus sensu lato* (*bcsl*) family that includes *Bacillus thuringiensis* and *B. anthracis*, includes a single *sleB* locus and two loci for *cwlJ*. The former is the first gene in a tri-cistronic operon that includes *ypeB* and *ylaJ*, a genetic organization that is generally observed in the *bcsl* family but not in other *Bacillus* species, where *ylaJ* is present elsewhere on the chromosome. Several studies have revealed a co-dependency between SleB and YpeB for their localization in the spore, although direct experimental evidence to support protein–protein interactions has not been obtained [17,20]. YlaJ is a member of the YlaJ/YhcN family of lipoproteins, which were shown recently to contribute to the stability and heat resistance of *B. subtilis* spores via an undefined role in the spore inner membrane (IM) [21]. Of the two *cwlJ* loci, the first (*cwlJ1*) is present as the first gene in a bi-cistronic operon with *gerQ*, as observed in other *Bacillus* species, whereas *cwlJ2* is mono-cistronic. The CwlJ1 and CwlJ2 proteins share 63% amino acid identity over 140 residues.

**Fig. 1.**
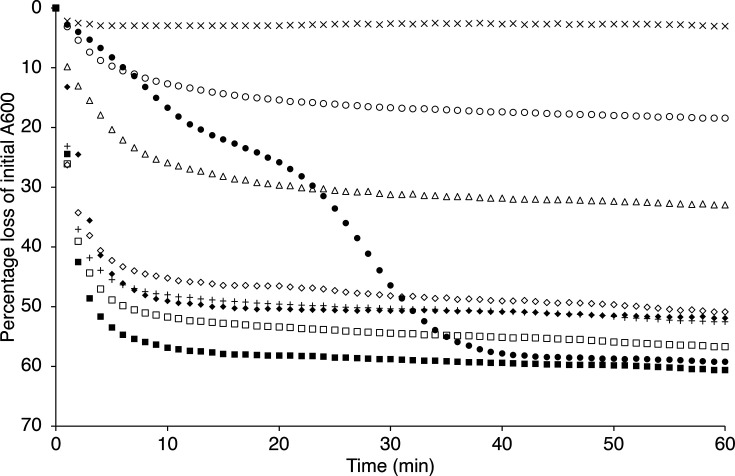
Germination of *B. cereus* CLE mutant spore suspensions. Spores of various strains were heat-shocked at 75 °C for 30 min and then cooled on ice before resuspending to an absorbance (600 nm) of 1.0 in buffer (10 mM Tris-HCl, pH 7.8) supplemented with 10 mM l-alanine plus 1 mM inosine. Absorbance measurements at 600 nm were recorded at 1 min intervals as described in the Methods section. Presented data are from single experiments, which are representative of analyses conducted with at least two independent batches of spores and where sd from mean values is <15%. Key: wild-type spores in buffer without germinants, ×; with germinants: wild type, filled squares; *sleB*, +; *cwlJ1*, filled diamonds; *cwlJ2*, open diamonds; *cwlJ1 cwlJ2*, closed circles; *sleB cwlJ1*, open triangles; *sleB cwlJ2*, open squares; *sleB cwlJ1 cwlJ2*, open circles.

Various single, double and triple CLE null mutant strains were constructed with a view to investigating the roles of these proteins in spore germination, using the markerless deletion approach that leaves just the first and last codon of the respective ORFs while minimizing potential polar effects on downstream genes (Table 1). The various strains were validated by PCR and sequencing and then assessed for colony-forming ability – often considered as a proxy for viability in spore germination studies – by plating and incubating defined aliquots of spores on LB medium overnight (Table 1). Germination characteristics of the spores were then tested as aqueous buffered suspensions supplemented with germinants (l-alanine and inosine) or CaDPA to assess the impact of the respective mutations on germination when induced via the respective germinant receptor (GR) and CwlJ-mediated systems (Figs 1 and S1).

Viability data for the various CLE mutants are broadly in line with those observed in other species [22], where loss of either of the CwlJ homologues has minimal, if any, effect on colony-forming ability. However, ~60% of spores lose the ability to form colonies when both *cwlJ1* and *cwlJ2* are deleted. Spores that are null for *sleB* show a similar loss of colony-forming ability. Deletion of *cwlJ1* in tandem with *sleB* results in a severe impairment to colony formation (~5-log reduction), which again is consistent with observations in other species. In contrast, deletion of *cwlJ2* in tandem with *sleB* does not impact upon viability any more than deletion of *sleB* alone, suggesting that of the CwlJ protein homologues, CwlJ1 is more important in terms of spore viability. If we consider germination as opposed to colony-forming ability, however, then CwlJ2 appears to be functional since the observed 30% reduction in absorbance in *sleB cwlJ1* spore suspensions is greater than that observed in *sleB cwlJ1 cwlJ2* triple mutant spore suspensions (~15% reduction in the latter is associated with CaDPA release and partial hydration of the core rather than appreciable cortex hydrolysis; Fig. 1). CLE single mutant spore suspensions all lose >50% A600, indicating significant cortex hydrolysis, although viability data suggest that this may not be sufficient in a significant fraction of *sleB* spores to proceed to outgrowth and colony-forming ability on LB medium. Also notable in Fig. 1 is the apparent delay in SleB activity in GR-mediated germination when it is the only functional CLE present (*cwlJ1 cwlJ2* spores). Data presented in Fig. S1 indicate that the *B. cereus* 10876 germinative response to endogenous CaDPA can be mediated via either CwlJ1 or CwlJ2, since only spores that lack both enzymes fail to respond to CaDPA. Deletion of *cwlJ1* results in a much slower loss in absorbance compared to *cwlJ2* null spores, again supporting the idea that the former plays a more significant role in cortex hydrolysis.

### Expression and localization of *B. cereus* 10876 CLEs

**Fig. 2.**
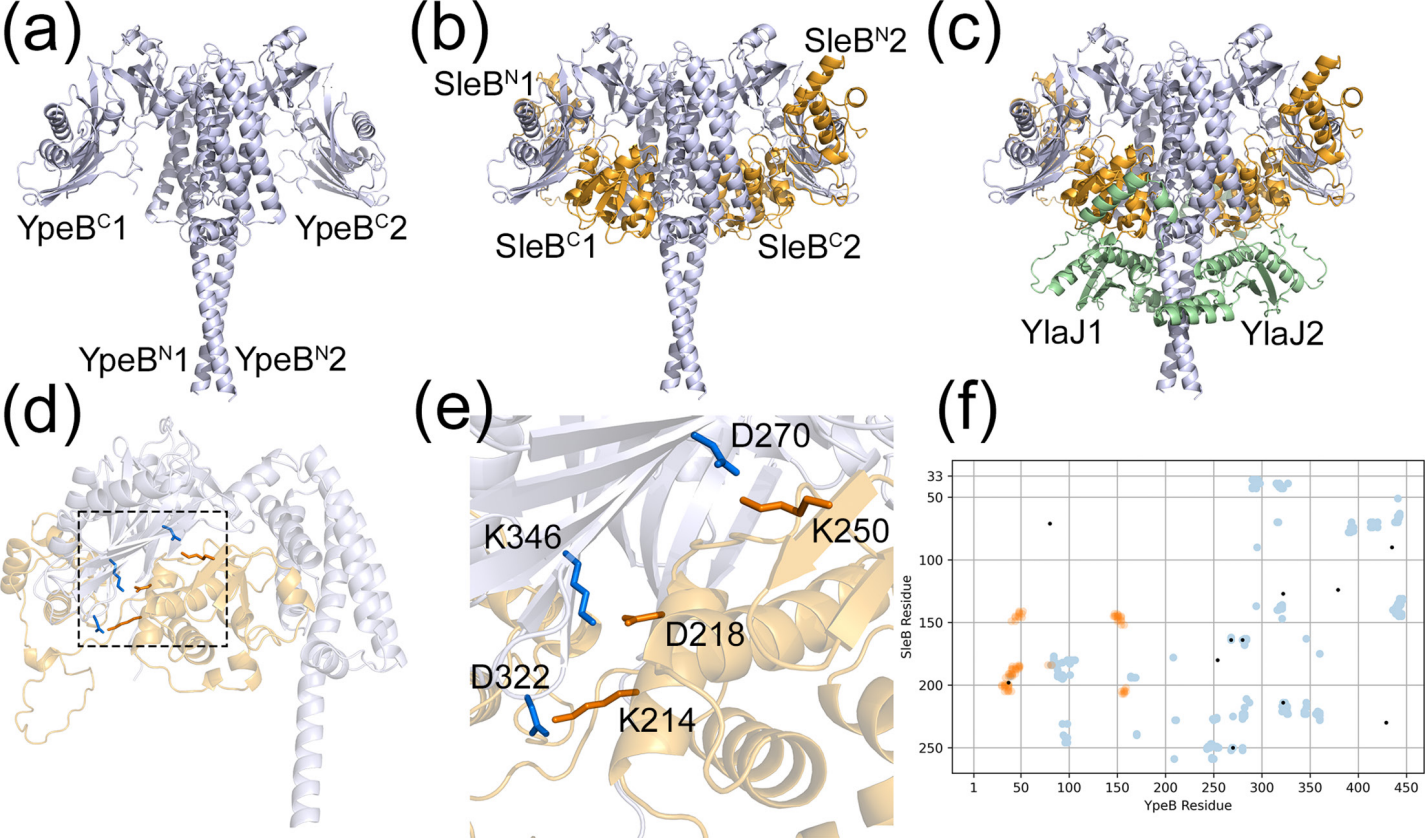
*B. cereus* SleB-YpeB-YlaJ complex. (**a**) AlphaFold 3 model of the predicted YpeB dimer. Hydrophobic residues within the vicinity of the N-terminal region of YpeB are expected to function as a lipid anchor to the spore IM (see also Fig. S5). (**b**) AlphaFold 3 model of the SleB-YpeB heterodimer. The catalytic site associated with the C-terminal domain of SleB is oriented towards the IM, whereas the N-terminal substrate-binding domain is oriented such that it can interact with spore peptidoglycan. (**c**) YlaJ lipoproteins are predicted to interact with YpeB’s N-terminal alpha helices, shielding SleB^C^, and anchoring the complex to the IM via lipidated cysteine residues located towards the N-terminus of YlaJ. Evolutionary covariance analysis identified a number of inter-protomer residues that are predicted to form contacts between proteins within the complex, including a number of salt bridges at the interface between SleB^C^ and YpeB^C^, as shown in the SleB-YpeB protomer in (**d**), and in more detail in (**e**). An EVcouplings scatter plot for SleB and YpeB is depicted in (**f**), where blue dots denote intra-protomer contacts, orange dots denote inter-protomer contacts and black dots denote evolutionary coupled residues.

**Fig. 3.**
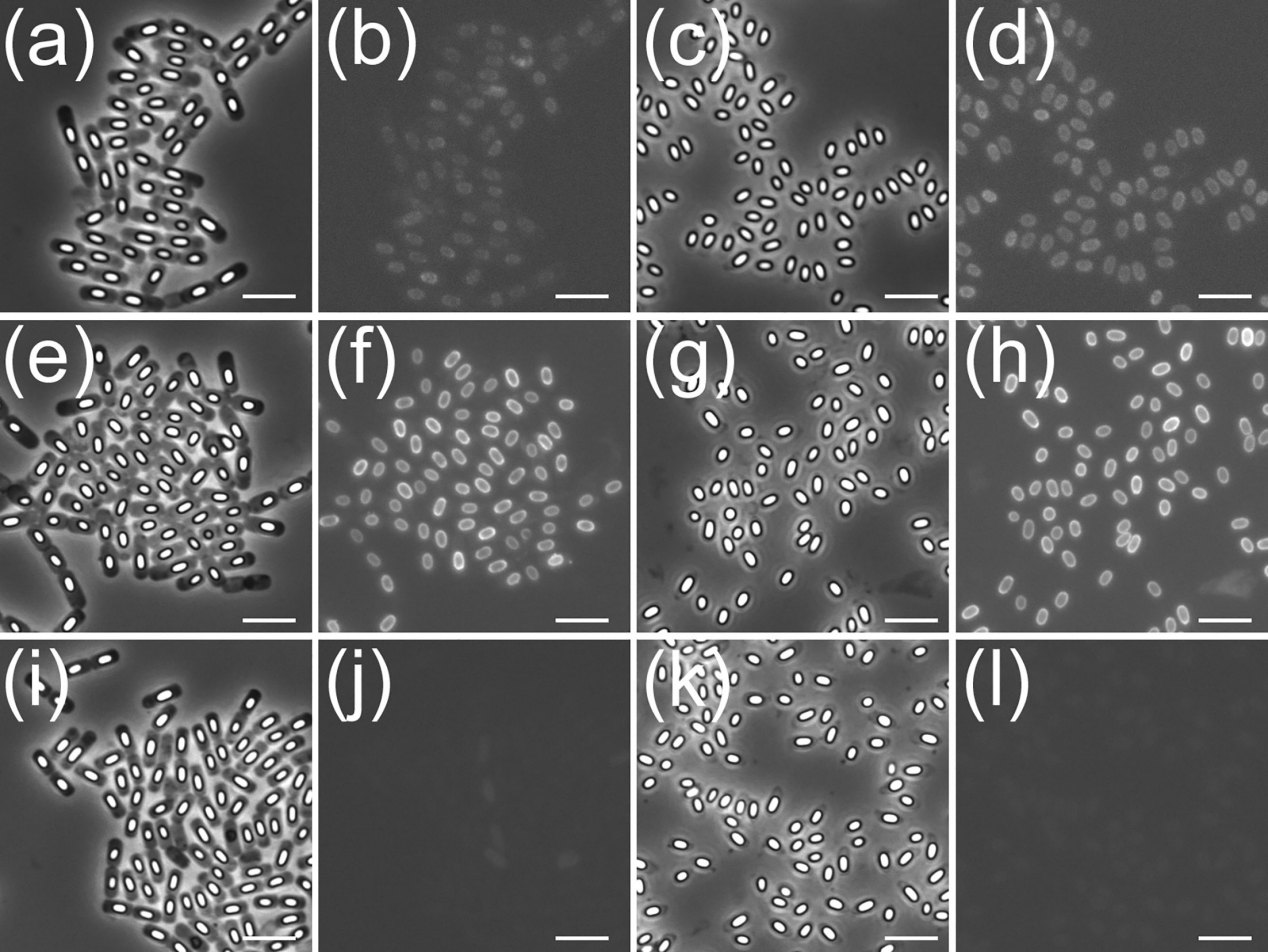
Phase-contrast and fluorescence microscopy images of *B. cereus* 10876 sporulating cells and mature spores with CLE fluorescent fusion proteins. Key: sporulating cells (**a, b**) and spores (**c, d**) with SleB^N^-tdTomato; sporulating cells (**e, f**) and spores (**g, h**) with CwlJ1-GFP; sporulating cells (**i, j**) and spores (**k, l**) with CwlJ2-GFP. Scale bar=5 µm.

A series of strains designed to express CLE fluorescent reporter fusion proteins under control of native regulatory sequences was constructed with a view to assessing the relative abundance and location of the various *B. cereus* CLEs within spores. Fluorophore fusions to full-length SleB (SleB^M^) or to the C-terminal catalytic domain (SleB^C^) failed to fluoresce (reasons for which may be inferred from the model presented in Fig. 2); hence, we were limited to working with the enzyme’s N-terminal substrate-binding domain (SleB^N^), i.e. using a genetic construct where the tdTomato ORF was placed immediately downstream of SleB’s N-terminal signal peptide sequence. We assumed that the latter was functional and was intended to result in transport of the protein across the IM to the mother cell side of the developing spore. The resultant fusion protein therefore comprised SleB^N^ with an N-terminal tdTomato fusion. Images of sporulating cells and mature spores of the SleB^N^-tdTomato, CwlJ1-GFP and CwlJ2-GFP strains are shown in Fig. 3. Fluorescence signal associated with CwlJ2-GFP was qualitatively less intense than that associated with CwlJ1-GFP, indicating that the former is expressed at lower levels. The relatively well-defined fluorescent ring is indicative of a coat location for CwlJ1 rather than IM, which is consistent with observations in *B. subtilis*. The radial locations of SleB^N^ and CwlJ1 were measured using the ELM technique, with mean values of 548.5 nm (±7.9 nm) for SleB^N^ and 566.0 nm (±3.1 nm) for CwlJ. Fluorescence intensity associated with CwlJ2 was insufficient to provide statistically robust ELM analyses. The difference between the mean radial locations of SleB^N^ and ClwJ1 (17.5 nm) may represent the thickness of the cortex if SleB is assumed to localize to the IM.

### Investigating SleB NTD’s role in enzyme localization

**Fig. 4.**
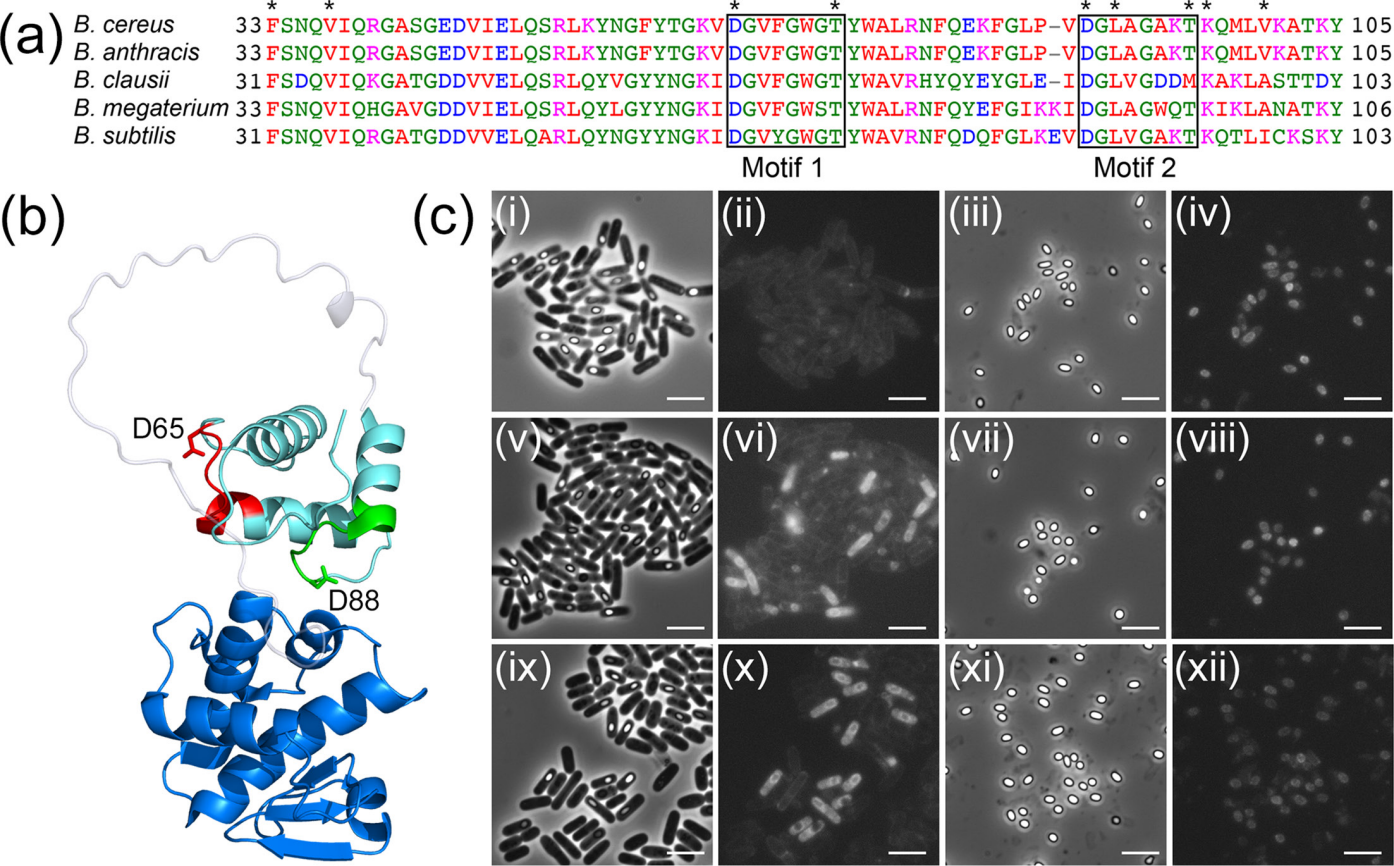
Structural and functional analysis of the N-terminal PG binding domain of SleB. (**a**) Sequence alignment of SleB from *Bacillus* spp. Numbers refer to the primary amino acid sequence with phenylalanine representing the first residue after cleavage of the signal peptide in the mature enzyme. Repeat sequence motifs 1 and 2 are boxed and labelled. Asterisks denote residues in *B. cereus* SleB^N^ that were subject to amino acid substitution in this work. (**b**) Crystal structures of the N- and C-terminal domains of *B. cereus* SleB, coloured cyan and blue, respectively, and oriented as per the AlphaFold model of the intact enzyme. Residues K104–Y142 form a loop that connects the two domains, shown in grey and modelled according to AlphaFold. The location of motif 1 is shown in red and motif 2 in green with functionally important D65 and D88 shown as sticks. (**c**) Phase-contrast and fluorescence microscopy images of *B. cereus gerE* sporulating cells and mature spores with plasmid-borne copies of *sleB^N^-tdTomato*. Key: sporulating cells (**i, ii**) and spores (iii, iv) with native SleB^N^; sporulating cells (**v, vi**) and spores (vii, viii) with SleB^N^ D65A; sporulating cells (ix, **x**) and spores (xi, xii) with SleB^N^ D88A. Scale bar=5 µm.

The most notable difference between SleB and CwlJ concerns the modular structure of the former, which comprises an N-terminal peptidoglycan binding domain (SleB^N^) that is joined by a linker sequence to the C-terminal catalytic domain (SleB^C^). CwlJ, in contrast, consists of a single domain, which is homologous to SleB^C^, i.e. despite fairly low sequence identity (23%) superposition of an AlphaFold model of *B. cereus* CwlJ with the SleB^C^ crystal structure (PDB: 4f55 [23]) reveals close structural alignment between the proteins (RMSD 0.92 Å for 70 Cα pairs). This includes the catalytic glutamate that occupies the same space in the structures of both SleB and CwlJ and which has been demonstrated to be essential to the lytic transglycosylase activity of SleB [23,24] and presumably CwlJ [19] (catalytic specificity has never been demonstrated unequivocally in the latter). However, SleB’s substrate binding domain is less well characterized in terms of structure function relationships. Having solved the crystal structure of *B. cereus* SleB^N^ several years ago (PDB: 6tci), and with appropriate null mutant and fluorescent reporter strains now available, we decided to attempt to redress the balance. The SleB^N^ domain comprises a three-helix bundle, which, based on comparative searches conducted with the *DALI* server [25], has a number of close structural neighbours, including the gp144 lytic transglycosylase peptidoglycan binding domain from the *Pseudomonas* bacteriophage φKZ (PDB:3bkh; RMSD 0.99 Å for 68 Cα pairs), an endolysin associated with the *Burkholderia* AP3 phage (PDB:5nm7; RMSD 0.86 Å for 61 Cα pairs) and *Clostridioides difficile* PDB entry 5tv7 (RMSD 0.80 Å for 61 Cα pairs). The C-terminal dimerization domain from the *B. subtilis* sporulation-specific CTPB protease is also a close structural homologue despite having a different functional role (PDB:4c2e; RMSD 0.74 Å for 60 Cα pairs [26]). Sequence analysis reveals two conserved repeat sequence motifs which are often present in other members of the pfam0147 family, particularly in Gram-positive bacterial lysins [27] (Fig. 4a). Motif 1 (D65 to T72) and motif 2 (D88 to T95) are observed in the SleB^N^ crystal structure to form surface-exposed grooves that could serve as potential substrate-binding sites (Fig. 4b). A number of residues that reside in these grooves (D65 and T72 from motif 1; D88, L90 and T95 from motif 2) and other residues of potential importance (F33, V37, K96 and V100, which contribute to a distinctly electropositive crevice) were identified and substituted for alanine by site-directed mutagenesis in the SleB^N^-tdTomato construct. The resultant plasmids encoding variant SleB^N^ proteins were introduced to the *gerE* null strain of *B. cereus* 10876. This strain has a coat defect such that any aberrant SleB^N^-tdTomato protein that fails to bind to the cortical peptidoglycan (PG) will diffuse away from the developing spore rather than being trapped by the coat [27,28]. The various strains were subject to analysis by microscopy throughout sporulation in liquid medium, which was observed to proceed normally with phase-bright spores developing within 48 h of inoculation. Fluorescence in most SleB^N^ variant strains observed a similar pattern to that observed with the native construct, i.e. a ring of fluorescence developing around the developing forespore sporangium that remained fluorescent in the mature spore. Two variant alleles – D65A and D88A – were associated with a different pattern of localization, in which the fluorescence signal was much more strongly dispersed throughout the entire mother cell during sporulation and then was observed only weakly – particularly in the D88A variant – in the mature spores (Fig. 4c). D65 and D88 represent the first residues in sequence motifs 1 and 2, respectively, with the crystal structure revealing that both are located on the perimeter of distinct surface depressions on either side of SleB^N^, where electrostatic and geometric factors presumably contribute to substrate interactions. As an aside, detection of SleB^N^-tdTomato-associated fluorescence in the mother cell is indicative of signal peptide recognition and transport of the fusion protein across the IM from the forespore.

Spores designed to express full-length SleB proteins with the D65A and D88A mutations, both individually and combined, from low-copy-number plasmids, were subsequently prepared in the CLE triple mutant background and subjected to germinative analysis by absorbance loss in the presence of l-alanine and inosine. The resultant data reveal that germination, and presumably cortex depolymerization, in spore suspensions with SleB D65A or D88A variant proteins proceeds at a similar rate to spores with the native protein (Fig. S2). Germination in suspensions of spores with the SleB D65A D88A double-variant protein is impacted negatively, however, with absorbance decreasing by just over 30%, versus 25% in the CLE null strain, and 50% in single-variant spores. Hence, despite the presence of the coat, it would appear that the bulk of the double-variant protein has not localized correctly during sporulation, perhaps by failing to interact with substrate. Alternatively, the introduction of two substitutions may have caused the protein to misfold, rendering it non-functional. Indeed, CD spectroscopy analyses conducted with purified recombinant SleB^N^ proteins bearing single (D65A or D88A) or combined (D65A/D88A) substitutions indicate that the variant proteins are to varying extents misfolded (Fig. S3), and this probably exerts a negative effect on germination.

### *B. cereus* YhcN and YlaJ lipoproteins

**Fig. 5.**
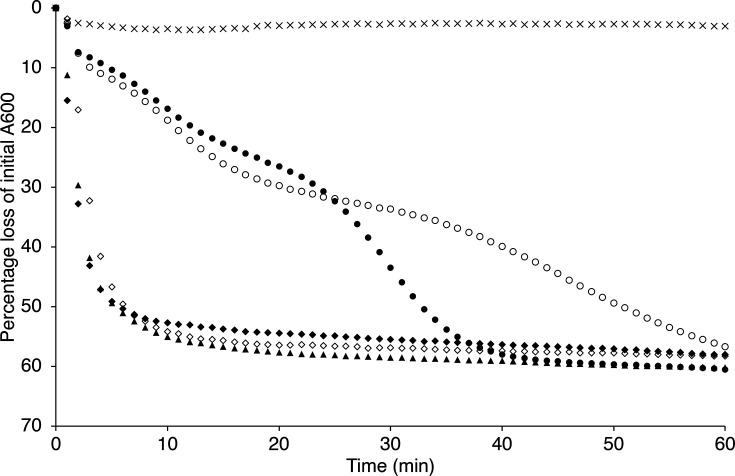
Germination of *B. cereus ylaJ* and *yhcN* null spore suspensions. Spores were heat-shocked at 75 °C for 30 min and then cooled on ice before resuspending to an absorbance (600 nm) of 1.0 in buffer (10 mM Tris-HCl, pH 7.8) supplemented with 10 mM l-alanine plus 1 mM inosine. Absorbance measurements at 600 nm were recorded at 1 min intervals as described in the Methods section. Presented data are from single experiments, which are representative of analyses conducted with at least two independent batches of spores and where sd from mean values is <10%. Key: wild-type spores in buffer without germinants, ×; wild-type spores in buffer with germinants, closed triangles; *sleB*, closed diamonds; *sleB ylaJ yhcN*, open diamonds; *cwlJ1 cwlJ2*, closed circle; *cwlJ1 cwlJ2 ylaJ yhcN*, open circles.

In common with *B. subtilis*, the *B. cereus* genome encodes four orthologues of the YlaJ/YhcN lipoprotein family proteins. These were demonstrated recently in *B. subtilis* to be semi-redundant and inter-changeable in a concentration-dependent manner and appear to have a role in establishing or maintaining the reduced fluidity of lipids in the spore IM [21]. In the course of this work, we created a number of *ylaJ* and *yhcN* null mutants in *B. cereus* 10876 with a view to assessing the impact, if any, on spore resistance and germination properties. These included *yhcN* and *ylaJ* single and double mutants in the parental strain, but also in otherwise isogenic strains where SleB or CwlJ were the only functional CLEs present. Spore viability data associated with deletion of *ylaJ* and *yhcN* individually and in tandem with various CLE mutations are detailed in Table 1. Deletion of *ylaJ*, the third gene in the *sleB ypeB* operon, resulted in a ~60% reduction in spore colony-forming ability, whereas the effect was less pronounced in this regard with *yhcN* null spores (~20% reduction in colony-forming ability). Deletion of both genes did not significantly affect colony formation compared to loss of *ylaJ* alone. The greatest reduction in colony-forming ability (~80%) was observed when both *yhcN* and *ylaJ* were deleted in addition to *cwlJ1* and *cwlJ2*. Overall, colony-forming data associated with lipoprotein null mutant spores are broadly in agreement with observations in *B. subtilis*, i.e. that loss of *yhcN*/*ylaJ* impacts negatively on SleB-associated cortex hydrolysis [29]. This assessment is borne out by absorbance-based germinative assays of spore suspensions of the various strains where deletion of *ylaJ* and/or *yhcN*, individually or when combined with deletion of *sleB*, results in absorbance loss profiles that are essentially indistinguishable from wild-type or *sleB* mutant profiles (Fig. 5). Similarly, deletion of *yhcN* in the *cwlJ1 cwlJ2* background, meaning SleB is the only functional CLE present, has no apparent effect on germination progression. However, deletion of *ylaJ* in the same background, or *ylaJ* and *yhcN*, results in an extended second stage of the spore population absorbance loss profile. Collectively, these data indicate that the YlaJ and YhcN lipoproteins exert little influence on CwlJ activity, but YlaJ, in particular, influences SleB activity during *B. cereus* spore germination. In contrast, when spores are germinated with the cationic detergent dodecylamine, which triggers germination by interacting with the IM in a thus far undefined manner, we see a reduction in the extent of absorbance loss in lipoprotein null spore suspensions, but not in a CLE-dependent manner (Fig. S4).

### SporeTracker analysis of *yhcN* and *ylaJ* null mutant spore germination

Absorbance measurements of spore suspensions are a relatively low-resolution approach to monitoring spore germination since they reflect the OD of a population of spores at a defined moment in time. By analysing a series of time-lapse micrographs containing tens to hundreds of spores as they transition from the phase bright to dark state when deposited on germinant-supplemented agar pads, the SporeTracker program permits the identification and quantitative assessment of the start, duration and end point of germination for multiple individual spores [13]. We decided to use this approach to examine the germination properties of spores of the *B. cereus* 14579 strain and isogenic null mutants since the corresponding 10876 strains had not been developed at the time of these experiments. Both strains are closely related within the convoluted *B. cereus sensu stricto* subgroup [30] but produce spores with differing morphologies and responses to defined germinants [31]. The resultant data reveal that initiation of germination under conditions used in SporeTracker experiments is generally slower than that observed when spores are suspended in aqueous germination buffer, presumably reflecting incubation on the microscope stage (~20 °C) and potential mass transfer limitations under the relatively immobilized agar pad conditions (Table 2). Regardless, the data reveal significant delays in the average time of initiation of germination in *ylaJ* and (in particular) *yhcN* null spores compared to the wild type. Similarly, initiation of germination in spores where SleB is the only functional CLE is extended considerably on average compared with wild-type spores (70 min versus 15 min). Deletion of *yhcN* or *ylaJ* extends this delay in initiating germination to over 120 min. Hence, bulk CaDPA release is delayed or impaired when SleB is the only major CLE, and the deletion of *yhcN* or *ylaJ* somehow compounds this defect. Notably, though, once started, the duration of germination – determined by SporeTracker as the time taken for pixel intensity to decrease from 90% to 10% of the range associated with spore phase transition – is comparable to wild-type spores, i.e. typically <10 min. Intriguingly, initiation and time to complete germination (using SporeTracker criteria) in spores where CwlJ1 and CwlJ2 are the only functional CLEs appear to be slightly more rapid than in wild-type spores (initiation circa 11 min, completion within 5 min). Deletion of *yhcN* and/or *ylaJ* has no impact on germinative rates of these spores.

**Table 2.** SporeTracker analysis of *B. cereus* 14579 CLE and lipoprotein null mutant spore germination*

Genotype	Average time (min)†		
	Start of germination‡	End of germination	Duration‡
Wild type	15.1±11.8	22.4±12.7	7.3±3.2
*ylaJ*	19.6±8.0**	28.9±9.2**	9.4±2.8**
*yhcN*	38.8±23.3**	55.8±24.2**	17.1±10.9**
*ylaJ yhcN*	30.3±16.1**	35.6±15.9**	5.3±1.7**
**SleB-dependent germination**			
*cwlJ1 cwlJ2*	70.5±20.7	78.9±21.1	8.4±3.1
*cwlJ1 cwlJ2 ylaJ*	135.0±33.3**	144.1±32.5**	9.1±3.2
*cwlJ1 cwlJ2 yhcN*	122.7±40.8**	132.1±41.6**	9.4±2.7**
*cwlJ1 cwlj2 ylaJ yhcN*	126.9±35.6**	134.6±35.4**	7.6±2.0
**CwlJ-dependent germination**			
*sleB*	11.3±12.7	16.0±13.1	4.7±1.7
*sleB ylaJ*	9.8±11.8	14.5±12.0	4.7±1.4
*sleB yhcN*	11.5±12.3	16.0±12.4	4.5±1.5
*sleB ylaJ yhcN*	12.9±13.5	17.1±13.6	4.2±1.2**

*Presented data are average values plus sd calculated from 100 spores of each variant, sampled from three independent biological replicates.

†Statistical analyses: ** indicates that the mean of the distribution between the mutant and wild type is significantly different (t-test, *P*<0.05).

‡Initiation of germination was determined when the pixel intensity in the centre region of marked spores fell below SporeTracker default threshold values. The time required for pixel intensity to drop from 90% to 10% of the entire drop range associated with spore phase transition was used to determine the duration of germination. Note that spores that remain phase bright for the duration of the experiment (typically <5%) are not included in the analyses.

### Structural model of a SleB-YpeB-YlaJ complex

One of the most enduring puzzles in the field of spore germination concerns the nature of SleB’s inactivity during dormancy and subsequent activation during germination. The interdependency between SleB and YpeB, where the absence by mutagenesis of one protein in the spore results in the mis-localization of the other, has led to a long-held hypothesis that these proteins interact to form a complex and that dissipation of this complex during germination results in SleB activity [2]. That the C-terminal domain of YpeB is composed of three sequential PepSY structural motifs that are associated with the inhibition of certain metallopeptidases adds an element of support, albeit tangentially, to the idea that the primary role of YpeB is to modulate SleB activity [32]. Regardless, experimental approaches designed to ascertain whether the proteins interact physically have, to date, either failed or provided only modest evidence that this is the case [19,20]. Given this impasse, we decided to adopt a structural bioinformatic approach, using the AlphaFold Multimer and, more recently, AlphaFold 3 structure prediction tools to ascertain whether SleB and YpeB might interact to form a plausible quaternary structural model. Furthermore, given its genetic and potential functional association with SleB [29], we used the same approach to query whether YlaJ might also form the third element of a larger SleB-YpeB-YlaJ complex.

The resultant favoured model from the various stoichiometric iterations tested, which is predicted with high confidence in terms of local and complex template-modelling scores, and which satisfies intermolecular interface contact criteria for reliable quaternary structure prediction [33], is shown in Fig. 2. The complex comprises two copies of each protein, arranged as a homodimer of heterotrimers with twofold rotational symmetry around a central axis formed by YpeB’s long N-terminal alpha helices. The most striking feature of the complex concerns the arrangement of SleB, which is held clamp-like between YpeB and YlaJ in each protomer. If we assume that the proximal hydrophobic region of YpeB’s N-terminal alpha helix serves as an IM anchor (Fig. S5), then catalytic SleB^C^ is oriented such that its active site is pointed towards the membrane and away from its cortical substrate. YlaJ is positioned underneath, its helical bundle running horizontally to the plane of the membrane and wrapped collar-like around YpeB’s N-terminal alpha helix. The net effect, presumably, is to hold SleB^C^ well away from its substrate during dormancy, while the remainder of the molecule traverses YpeB’s PepSY-containing C-terminal domain, leaving SleB^N^ free to bind to the germ cell wall or cortex. At the same time, the lipidated N-terminal region of YlaJ should provide further anchorage to the membrane, adding to the stability of the entire complex.

**Fig. 6.**
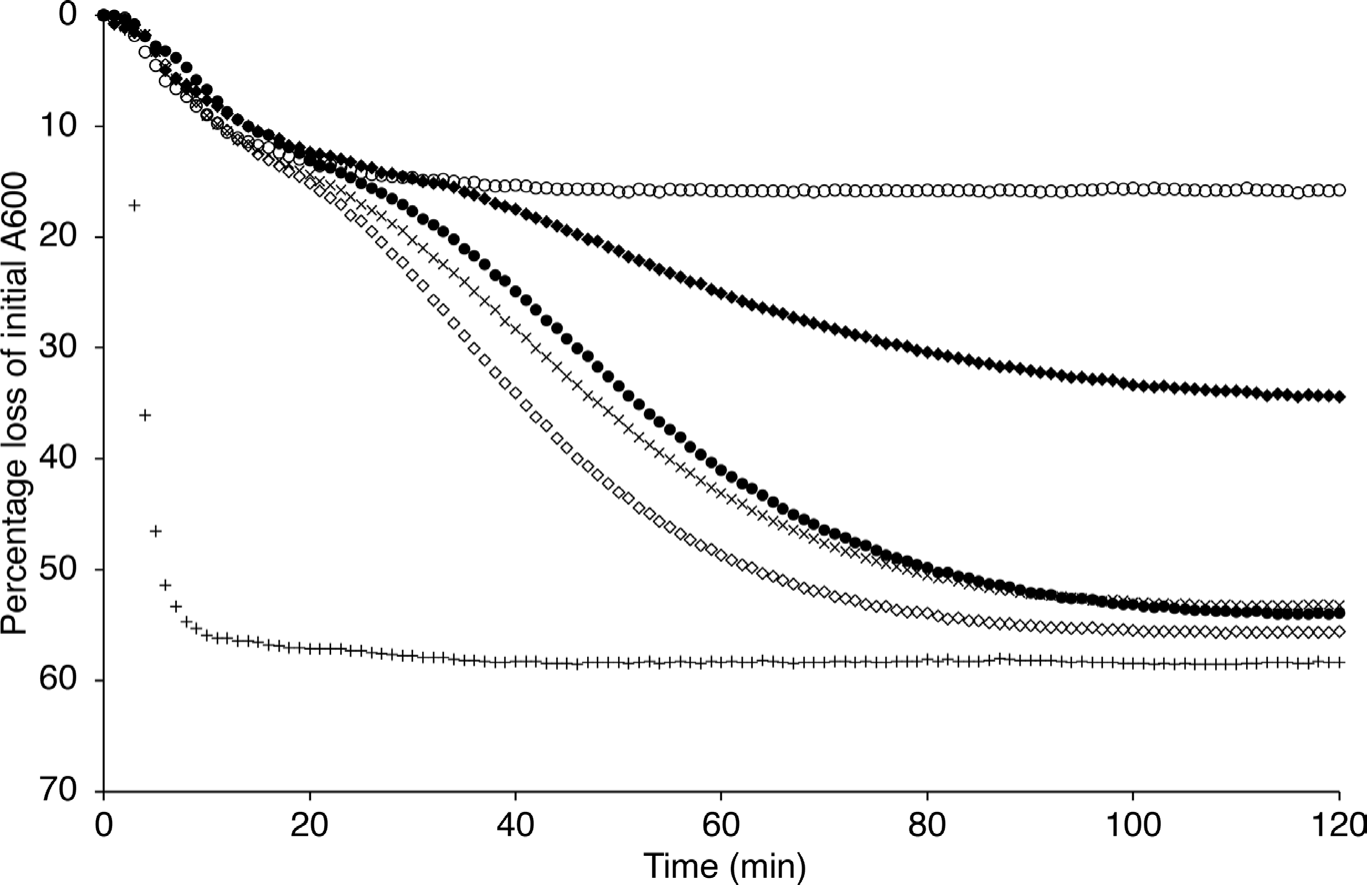
Germination of suspensions of *B. cereus* spores with mutations designed to disrupt ionic bonds between defined SleB and YpeB residues. *B. cereus CLE3* mutant spores complemented with plasmid-borne variant *sleB* and/or *ypeB* genes were heat-shocked at 75 °C for 30 min and then cooled on ice before resuspending to an absorbance (600 nm) of 1.0 in buffer (10 mM Tris-HCl, pH 7.8) supplemented with 10 mM l-alanine plus 1 mM inosine. Absorbance measurements at 600 nm were recorded at 1 min intervals as described in the Methods section. Presented data are from single experiments, which are representative of analyses conducted with at least two independent batches of spores and where sd from mean values is <12%. Key: wild-type spores, + symbols; CLE3 spores with native SleB and YpeB, closed circles; SleB K250A, × symbols; YpeB D322A D270A, open diamonds; SleB D218A YpeB D322A, closed diamonds; SleB D218A YpeB D322A D270A, open circles. CLE3 mutant data is not shown for clarity but overlays SleB D218A YpeB D322A D270A data points. SleB K250A data are representative of other strains with single alanine substitutions to SleB or YpeB (Table 1), which are omitted for clarity.

However, whilst the AlphaFold model is undoubtedly visually and conceptually appealing, an obvious question concerns the extent to which it reflects reality. Evolutionary coupling analysis (ECA) is useful in this regard, at least as a prelude to more detailed experimental structural analyses, permitting, as it does, the identification of pairs of residues that have co-evolved in protein tertiary or quaternary structures in order to preserve the stability of their interactions [34]. ECA in this case supports the predicted homodimer-of-heterotrimers model for the SleB-YpeB-YlaJ complex since there is a high coincidence between numerous evolutionary couplings and points of intra-protomer contact within the complex (Fig. S6 and Tables S3–S7). These include a number of potentially stabilizing salt bridges between SleB^C^ and YpeB^C^ (SleB K214–YpeB D322, SleB D218–YpeB K346 and SleB K250–YpeB D270), and, for example, between YpeB and YlaJ (YpeB K23–YlaJ R90, YpeB Y19–YlaJ L142 and YpeB K23–YlaJ D143; Fig. 5c, d). ECA additionally identifies a number of residues predicted to form inter-protomer contacts, predominantly between the N-terminal regions of YpeB, and which lends credence to the AlphaFold model. As a preliminary test of the structural model, a series of constructs designed to disrupt predicted contacts formed by salt bridges between SleB and YpeB were prepared by site directed mutagenesis (SDM), i.e. introducing alanine substitutions at the following SleB K214, D218 and K250 residues and the corresponding D322, K346 and D270 locations in YpeB. Low-copy-number plasmids encoding the variant proteins were introduced to the *B. cereus* CLE3 strain and the resultant spores tested for germination by absorbance loss and viability on LB plates. Outputs from these analyses revealed that disruption to individual salt bridges via alanine substitutions at any of the SleB or YpeB residues of interest exerted negligible effects on spore properties compared to the CLE3 strain complemented with native *sleB* and *ypeB* (Fig. 6). Germination-associated absorbance loss was slightly faster than control spores in one of the strains with two disrupted salt bridges (YpeB D322A D270A), whereas the strain with SleB D218A YpeB D322A substitutions showed an impaired absorbance response and a defect in colony-forming ability (~10% of spores formed colonies on LB medium). Absorbance loss attributable to cortex hydrolysis was completely diminished in the strain with disruptions to all three SleB YpeB salt bridges of interest (SleB D218A YpeB D322A D270A), which was reflected also in spore colony-forming ability commensurate with the CLE3 strain (0.002%). Whether the apparent loss of SleB function during germination is a consequence of the cumulative loss of salt bridges weakening interactions within the SleB-YpeB complex, resulting in protein mislocalization and/or proteolysis during sporulation, has not unequivocally been established and will require further analysis. However, loss of activity due to misfolding of either SleB or YpeB may not necessarily be the case since strains with SleB D218A and YpeB D322A D270A substitutions germinated normally.

## Discussion

Work described in this manuscript explores various aspects of the second major stage of spore germination in *B. cereus*, i.e. cortex depolymerization, from which a number of observations can be made. First, as anticipated from studies in other species, the SleB and CwlJ CLEs are semi-redundant in the sense that they can partially compensate in spores for the absence of the other, depolymerizing the cortex to an extent that supports colony-forming ability in significant fractions of the spore population (with CwlJ2 being a contributing but minor player in this regard). Hence, SleB and CwlJ’s complementary functions are almost certainly genus-wide, and any CLE-focused attempt at controlling spores should probably target both enzymes while also considering potential confounding factors in the shape of additional homologues.

A second observation concerns structural insight to the role of SleB’s PG binding domain. Sequence motifs first recognized several decades ago [27,35] can now be visualized in the crystal structure of the *B. cereus* enzyme and the importance of the conserved aspartate residues in each motif identified from SDM and germination experiments. However, the precise role of these motifs in the SleB^N^ domain’s interaction with PG – and indeed whether the domain interacts with structurally distinct cortical PG and/or germ cell wall PG within the spore – has yet to be determined and will require further investigation.

The fate of YlaJ, the third protein encoded in the SleB operon, is intertwined with the final series of observations associated with this work. The nature of the putative interaction between SleB and YpeB, how YlaJ might fit into this scheme [29] and how these proteins collectively determine SleB’s inactivity during spore dormancy and subsequent activation during germination has been scrutinized since SleB’s initial purification from spore extracts in the 1980s [36,37]. Molecular genetic approaches have revealed an interdependence between the proteins, to varying degrees, while biochemical and structural approaches have revealed detail in terms of the catalytic bond specificity of the enzyme and hints of how YpeB might interact with the former. Unfortunately, the crystal structure inventory for these proteins is incomplete [23,24, 32, 38], particularly in terms of co-crystal structures between individual proteins and the absence of holoenzyme information. Hence, the availability of AlphaFold models of the SleB-YpeB-YlaJ complex, predicted with high confidence levels for the *B. cereus* proteins, but also for other species (data not shown), and supported further by evolutionary coupling analyses, is potentially significant. That the model readily yields clues as to how SleB activity is suppressed during dormancy – displaced and shielded from the cortical substrate – demonstrates the value, once again [3], of the computational approach when applied to long-standing questions concerning spore germination. Equally, the model has regions that are predicted with less certainty; hence, the challenge moving forward will be to design and conduct appropriate experimental approaches that go beyond the mutagenesis and EC analyses reported here to further validate and refine the model. *In situ* crosslinking and immunoprecipitation or co-crystallization experiments may be useful in this regard. Likewise, while the AlphaFold model is a good fit, conceptually, with the dormant spore, insight to the molecular mechanisms that lead to the presumed destabilization of the complex during germination – perhaps associated with changes in membrane tension or fluidity that occur after GR and SpoVA mediated ion fluxes – and ultimately to activation of SleB, represents a major area for future work.

## Supplementary material

10.1099/mic.0.001591 Uncited Supplementary Material 1.

## Abbreviations

A600: absorbance at 600 nm
CLEs: cortex lytic enzymes
ECA: evolutionary coupling analysis
ELM: ellipsoid localization microscopy
GR: germinant receptor
IM: inner membrane
LB: Lysogeny broth
PG: peptidoglycan
SDM: site directed mutagenesis
SNB: supplemented nutrient broth
