# Supplementary material for: Structural and functional analysis of Bacillus cereus spore cortex lytic enzymes and YlaJ/YhcN lipoproteins
Source: Microbiology (Reading). 2025 Aug 7;171(8):001591. doi: 10.1099/mic.0.001591 (PMC12331253; doi:10.1099/mic.0.001591)
Supplement: Uncited Supplementary Material 1. [file mic-171-01591-s001.pdf]

Supplemental Information

Structural and functional analysis of *Bacillus cereus* spore cortex lytic enzymes and YlaJ/YhcN lipoproteins

Amin Mustafa, Bahja Al-Riyami, Giannina Ow-Young-Villareal, Rebecca Caldbeck, David Chan Hian Pin, Joshua Yarrow and Graham Christie

This file includes:

Supplemental Tables 1 - 11
Supplemental Figures 1 – 6

Table S1 *Bacillus cereus* 14579 and isogenic derivative strains used in this work and colony forming ability of their spores[a]

Strain	Genotype	Colony forming ability (%)[b]	Source (reference)
14579	Wild type	100	Toril Lindback
Null mutant strains			
AM004	*ΔylaJ*	50	This work
AM005	*ΔyhcN*	74	This work
AM006	*ΔylaJ ΔyhcN*	47	This work
AM007	*ΔcwlJ1 ΔcwlJ2*	84	This work
AM008	*ΔcwlJ1 ΔcwlJ2 ΔylaJ*	71	This work
AM009	*ΔcwlJ1 ΔcwlJ2 ΔyhcN*	59	This work
AM010	*ΔcwlJ1 ΔcwlJ2 ΔylaJ ΔyhcN*	65	This work
AM013	*ΔsleB*	51	This work
AM014	*ΔsleB ΔylaJ*	49	This work
AM015	*ΔsleB ΔyhcN*	54	This work
AM016	*ΔsleB ΔylaJ ΔyhcN*	56	This work

[a] Spores of various strains were prepared, purified, and their ability to form colonies on LB medium determined as described in Materials and Methods. All values shown are averages of results with two independent spore preparations and are ±25% of the value shown.

[b] Colony forming ability of wild type spores was set at 100%.

Table S2 NCBI Accession Identifiers

Protein[a]	Accession ID	
	B. cereus 10876	*B. cereus* 14579
SleB	EEK50464	AAP09706
YpeB	EEK50463	AAP09705
YlaJ	EEK50462	AAP09704
CwlJ1	EEK47671	AAP12252
CwlJ2	EEK50637	AAP09496
YhcN	EEK48698	AAP11332
YutC	EEK48093	AAP11839
CoxA	EEK47968	AAP12011

[a] Named as per *B. subtilis* orthologues

Tables S3 – S7 EVCouplings for SleB-YpeB, YpeB-YlaJ and YpeB-YpeB

Table S3 SleB-YpeB Coupled Contacts (Probability Threshold 0.6)

	SleB	YpeB	Probability (2 d.p.)	Contact Type
1	LEU 164	TRP 280	1.00	Intra-Protomer
2	LEU 164	TYR 268	1.00	Intra-Protomer
3	LYS 214	ASP 322	1.00	Intra-Protomer
4	LYS 250	ASP 270	0.98	Intra-Protomer
5	ASP 218	LYS 346	0.72	Intra-Protomer
6	ALA 180	ALA 105	0.62	Intra-Protomer

Table S4 YpeB-YlaJ Coupled Contacts (Probability Threshold 0.6)

	YpeB	YlaJ	Probability (2 d.p.)	Contact Type
1	LYS 23	ARG 90	1.00	Intra-Protomer
2	TYR 19	LEU 142	0.95	Intra-Protomer
3	LYS 23	ASP 143	0.63	Intra-Protomer

Table S5 SleB-YpeB All Couplings (Probability Threshold 0.8)

	SleB	YpeB	Probability (2 d.p.)	Contact Type
1	LEU 164	TRP 280	1.00	Intra-Protomer
2	ALA 180	TYR 254	1.00	None
3	LEU 164	TYR 268	1.00	Intra-Protomer
4	LYS 214	ASP 322	1.00	Intra-Protomer
5	LYS 250	ASP 270	0.98	Intra-Protomer
6	LEU 90	VAL 435	0.93	None
7	LEU 230	SER 429	0.92	None
8	GLY 71	THR 80	0.88	None
9	THR 124	ALA 379	0.86	None
10	ALA 198	ASN 37	0.84	None
11	GLU 127	ASP 322	0.84	None

Table S6 YpeB-YlaJ All Couplings (Probability Threshold 0.8)

	YpeB	YlaJ	Probability (2 d.p.)	Contact Type
1	LYS 23	ARG 90	1.00	Intra-Protomer
2	LYS 23	GLN 131	1.00	None
3	LYS 23	GLN 127	0.99	None
4	LYS 148	GLU 183	0.99	None
5	ILE 5	ASP 89	0.99	None
6	TYR 268	ALA 147	0.98	None
7	GLU 27	ALA 147	0.98	None
8	HIS 308	ASP 89	0.98	None
9	GLY 198	ALA 101	0.98	None
10	VAL 31	THR 121	0.97	None
11	HIS 366	ILE 79	0.97	None
12	MET 95	GLU 102	0.96	None
13	ILE 33	ALA 61	0.95	None
14	LEU 154	VAL 34	0.95	None
15	TYR 19	LEU 142	0.95	Intra-Protomer
16	LYS 23	GLU 94	0.94	None
17	LYS 155	GLU 180	0.93	None
18	GLU 186	VAL 116	0.92	None
19	GLU 126	GLU 180	0.91	None

20	ASP 307	HIS 195	0.91	None
21	PHE 424	LYS 47	0.90	None
22	LYS 148	LEU 145	0.90	None
23	LYS 257	VAL 39	0.90	None
24	PHE 231	ALA 78	0.90	None
25	ASN 125	VAL 63	0.89	None
26	ALA 74	VAL 156	0.89	None
27	VAL 391	ASP 89	0.87	None
28	VAL 330	VAL 63	0.86	None
29	LYS 23	ALA 147	0.84	None
30	LYS 375	ASN 189	0.84	None
31	ASN 141	ASP 190	0.84	None
32	VAL 8	GLU 181	0.83	None
33	TYR 329	ASN 67	0.83	None
34	CYS 409	ALA 101	0.83	None
35	GLN 26	ASP 173	0.81	None

Table S7 YpeB-YpeB Inter-Protomer Coupled Contacts (Probability Threshold 0.8, Short-Range Included)

	**YpeB 1**	**YpeB 2**	**Probability (2 d.p.)**	**Contact Type[a]**
1	THR 17	GLY 18	1.00	Multi
2	ARG 41	GLN 89	1.00	Multi
3	TRP 20	GLY 21	1.00	Multi
4	ASP 55	LYS 437	1.00	Inter-Protomer
5	GLU 24	HIS 25	1.00	Multi
6	GLY 58	TYR 421	1.00	Inter-Protomer
7	VAL 14	THR 17	1.00	Multi
8	SER 67	ALA 431	1.00	Inter-Protomer
9	ALA 74	ILE 116	1.00	Inter-Protomer
10	GLY 21	GLU 24	1.00	Multi
11	ILE 6	ILE 7	1.00	Multi
12	GLU 27	LYS 28	1.00	Multi
13	ILE 7	LEU 10	0.99	Multi
14	GLU 24	LYS 28	0.97	Multi
15	LEU 10	THR 11	0.97	Multi
16	GLU 82	SER 85	0.96	Multi
17	ALA 62	LEU 416	0.95	Inter-Protomer
18	GLY 13	VAL 14	0.94	Multi
19	SER 81	GLU 82	0.92	Multi
20	VAL 31	LEU 32	0.90	Multi
21	ASN 64	PRO 387	0.82	Inter-Protomer
22	TRP 20	TYR 22	0.81	Multi

[a] Multi refers to both inter-protomer and intra-protomer contacts; short-range couplings, usually omitted, are within 5 amino acids in the primary structure.

Table S8 Oligonucleotide primers used to create *B. cereus* 10876 null mutant strains

Strain genotype	Primer	Sequence (5' to 3')
Δ*sleB*	pMAD-500up-*sleB*-For	CCATGGTACCCGGGAGCTCGAATTCCCCCACTGATTAAGGTTTCACTTTA
	500up-*sleB*-Rev	ACATTGTCCCACCTCCGCTTTACATAATTTTCCCTCCTTAATTACGGAAA
	500down-*sleB*-For	AAATCATTTACATAATTAAATGTAAGGAGGTGGGACAATGTTACGAGGTA
	500down-*sleB*-Rev	CCTCGCGTCGGGCGATATCGGATCCACATCCATCCAGCGTAAATTATTTT
Δ*cwlJ1*	pMAD-500up-*cwlJ1*-For	CCATGGTACCCGGGAGCTCGAATTCAAAGGGAAAGAAGACCAAGTAACTG
	ATG-500up-*cwlJ1*-Rev	CCCTCCTCATTTTTATTCCTTACATTATGTGTCACCTCTCTTTTTGGAAT
	ATG-500down-*cwlJ1*-For	AAAAGAGAGGTGACACATAATGTAAGGAATAAAAATGAGGAGGGATTTTT
	pMAD-500down-*cwlJ1*-Rev	CCAGCCTCGCGTCGGGCGATATCGGATCCAAAAGGATTATAAAAAAACAAAAGCTGG
Δ*cwlJ2*	pMAD500up-*cwlJ2*-For	CCATGGTACCCGGGAGCTCGAATTCACATGTTCGTTATGTATTGGGACAA
	ATG-500up-*cwlJ2*-Rev	ACTATAACAGAAAGGTGGTATCACTATGTAATAAAAAAGAAAACACCGAA
	ATG-500Down-*cwlJ2*-For	ACAGAAAGGTGGTATCACTATGTAATAAAAAAGAAAACACCGAAAGTACC
	pMAD-500down-*cwlJ2*-Rev	CCTCGCGTCGGGCGATATCGGATCCTAAAATTGTACCCTCTTCAACGGTT
Δ*ylaJ*	pMAD-500up-*ylaJ*-For	CCATGGTACCCGGGAGCTCGAATTCCAGAAAGTTAGTTTAAACGATGCAGG
	ATGTAA-500up-*ylaJ*-Rev	ACGAGGCTAATTTTACATTATTTTCACCACTTCACTATTAAGGTTTAATC
	ATGTAA-500down-*ylaJ*-For	AGTGAAGTGGTGAAAATAATGTAAAATTAGCCTCGTACTTAATTGTACGA
	pMAD-500down-*ylaJ*-Rev	CCTCGCGTCGGGCGATATCGGATCCTGTATGTTCATGCTAAACTCTCCCA
Δ*yhcN*	pMAD-500up-*yhcN*-For	CCATGGTACCCGGGAGCTCGAATTCTCAACAACCAAATCCATATCAAATG
	ATGTAA-500up-*yhcN*-Rev	CCCTTCCTCTTTTGTTTTGTTACATTACAAAAACCCCCTCTGTTAGAATG
	ATG-500Down-*yhcN*-For	AACAGAGGGGGTTTTTGTAATGTAACAAAACAAAAGAGGAAGGGGGAGCC
	pMAD-500down-*yhcN*-Rev	GCCTCGCGTCGGGCGATATCGGATCCAGCGAAATTATAATAAACAGGAGATGG
Δ*gerE*	pMAD500up-*gerE*-For	CCATGGTACCCGGGAGCTCGAATTCTAATGGCATGTGCATTACTAGTCGT
	ATG-500up-*gerE*-Rev	ATAAAAGTCGGCTGCTTTCTTACATCTGGTATAACCCTCCTTGCTTAAGC
	ATG-500Down-*gerE*-For	GCAAGGAGGGTTATACCAGATGTAAGAAAGCAGCCGACTTTTATATAAAA
	pMAD-500down-*gerE*-rev	CCTCGCGTCGGGCGATATCGGATCCAAAGAGCTTACTCCGTGACAAACAG

Table S9 Oligonucleotide primers used to create fluorescent fusion constructs

Strain genotype	Primer	Sequence (5' to 3')
P*sleB-sleB^{N}-tdTomato*	pHT *sleB* For	ctatgaccatgattacgccaagcttccccactgattaaggtttcacttta
	sleB^{N} tdTom Rev	ccttttgctgctgcttctccgcctcctgacttgtcatactttgttgctttcacgagcatt
	sleB^{N} tdTom For	ggaggcggagaagcagcagcaaaaggcggaggcgttagcaagggtgaagaagttataa
	pHT *tdTom* Rev	ttgtaaaacgacggccagtgaattcttacttataaagttcatccattcca
P*cwlJ1-cwlJ1-gfp*	pHT *cwlJ1* For	ctatgaccatgattacgccaagcttaaaagctggctcacgctagcttttt
	cwlJ1 gfp Rev	cttttgctgctgcttctccgcctccatatacgctagggcagtctccagca
	cwlJ1 gfp For	ggaggcggagaagcagcagcaaaaggcggaggcagtaaaggagaagaacttttcactg
	pHT *gfp* Rev	gttgtaaaacgacggccagtgaattcttatttgtatagttcatccatgcc
P*cwlJ2-cwlJ2-gfp*	pHT *cwlJ2* For	ctatgaccatgattacgccaagcttacatgttcgttatgtattgggacaa
	cwlJ2 gfp Rev	cttttgctgctgcttctccgcctcctcgtgaatacattttcggacattca
	cwlJ2 gfp For	ggaggcggagaagcagcagcaaaaggcggaggcagtaaaggagaagaacttttcactg
	pHT *gfp* Rev	gttgtaaaacgacggccagtgaattcttatttgtatagttcatccatgcc
P*sleB-sleB-ypeB-ylaJ-tdTomato*	pHT *sleB* For	ctatgaccatgattacgccaagcttccccactgattaaggtttcacttta
	ylaJ tdTom Rev	cttttgctgctgcttctccgcctcctttattcaaatgattatttgattgatcattctgtt
	ylaJ tdTom For	ggaggcggagaagcagcagcaaaaggcggaggcgttagcaagggtgaagaagttataa
	pHT *tdTom* Rev	ttgtaaaacgacggccagtgaattcttacttataaagttcatccattcca
P*yhcN-yhcN-tdTomato*	pHT *yhcN* For	ctatgaccatgattacgccaagctttcaacaaccaaatccatatcaaatg
	yhcN tdTom Rev	cttttgctgctgcttctccgcctcctcgcactgtaccagttaaaccgtaa
	yhcN tdTom For	ggaggcggagaagcagcagcaaaaggcggaggcgttagcaagggtgaagaagttataa
	pHT *tdTom* Rev	ttgtaaaacgacggccagtgaattcttacttataaagttcatccattcca

Table S10 Oligonucleotide primers used for $SleB^N$ SDM experiments

Subtitution	Sequence (5' to 3')
F33A	tatacaactaaagaatgtagaagctgcctctaatcaagtcattcaaagag
	tgaatgacttgattagaggcagcttctacattctttagttgtatactact
V37A	atacaactaaagaatgtagaagctttttctaatcaagccattcaaagagg
	atgcccctctttgaatggcttgattagaaaaagcttctacattctttagt
D65A	ggtgttttcggatggggtacatattg
	gaaaacaccggccacttttcccgtataaaatccgttat
T72A	atggtgttttcggatggggtgcctattgggcacttcggaattttcaagag
	gaaaattccgaagtgcccaataggcaccccatccgaaaacaccatccact
D88A	agagaaattcggattacccgttgccggtttagctggagctaaaacgaagc
	tcgttttagctccagctaaaccggcaacgggtaatccgaatttctcttga
L90A	attcggattacccgttgatggtgccgctggagctaaaacgaagcaaatgc
	tttgcttcgttttagctccagcggcaccatcaacgggtaatccgaatttc
T95A	tgatggtttagctggagctaaagccaagcaaatgctcgtgaaagcaacaa
	ttgctttcacgagcatttgcttggctttagctccagctaaaccatcaacg
K96A	tggtttagctggagctaaaacggcccaaatgctcgtgaaagcaacaaagt
	ggccgttttagctccagctaaaccatca
V100	ctcgccaaagcaacaaagtatgacaagtcaggaggcggagaagcagcagcaaaag
	ccttttgctgctgcttctccgcctcctgacttgtcatactttgttgctttggcgagcatt

Table S11 Oligonucleotide primers used for SleB-YpeB complex SDM experiments

Subtitution	Sequence (5' to 3')
SleB K214A	ccgatggacaaatatatttaacaccaaatgaaacagcaaaagctgcagttttagatgcgattaac
	gttaatcgcatctaaaactgcagcttttgctgtttcatttggtgttaaatatatttgtccatcgg
SleB D218A	acagcaaaaaaagcagttttagctgcgattaacggatggga
	tcccatccgttaatcgcagctaaaactgctttttttgctgt
SleB K250A	agtaaatggatttggactcgtccacaaattgcaaaaatcggtaaacatattttctg
	cagaaaatatgtttaccgatttttgcaatttgtggacgagtccaaatccatttact
YpeB D270A	gaacaatgagttctatatggctattaccggaaaaggcggat
	atccgccttttccggtaatagccatatagaactcattgttc
YpeB D322A	gctttatgatagctctcaatatgctaatgttggtgtatttacgtatg
	catacgtaaatacaccaacattagcatattgagagctatcataaagc
YpeB K346A	acgaatttatccagaagcaatccaaatggctattgctttagatgatggttctatcg
	cgatagaaccatcatctaaagcaatagccatttggattgcttctggataaattcgt

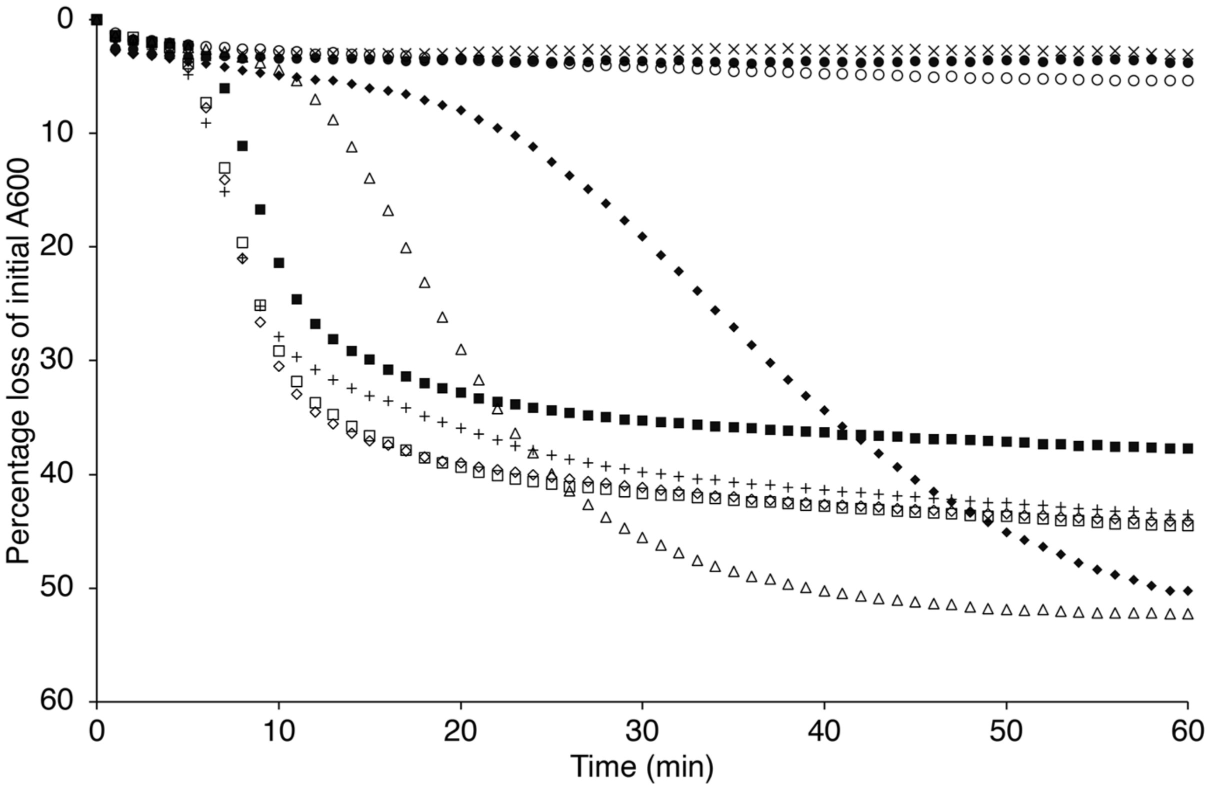

Figure S1 CaDPA mediated germination of *Bacillus cereus* CLE mutant spores. Spores of various strains were resuspended to an absorbance (600 nm) of 1.0 in buffer (10 mM Tris-HCl, pH 7.8) supplemented with 60 mM CaDPA and the absorbance at 600 nm recorded at 1 minute intervals. Presented data are from single experiments, which are representative of analyses conducted with at least two independent batches of spores and where SD from mean values is <10%. Key: wild type spores in buffer without germinants, ×; with germinants: wild type, filled squares; *sleB*, +; *cwlJ1*, filled diamonds; *cwlJ2*, open diamonds; *cwlJ1 cwlJ2*, closed circles; *sleB cwlJ1*, open triangles; *sleB cwlJ2*, open squares; *sleB cwlJ1 cwlJ2*, open circles.

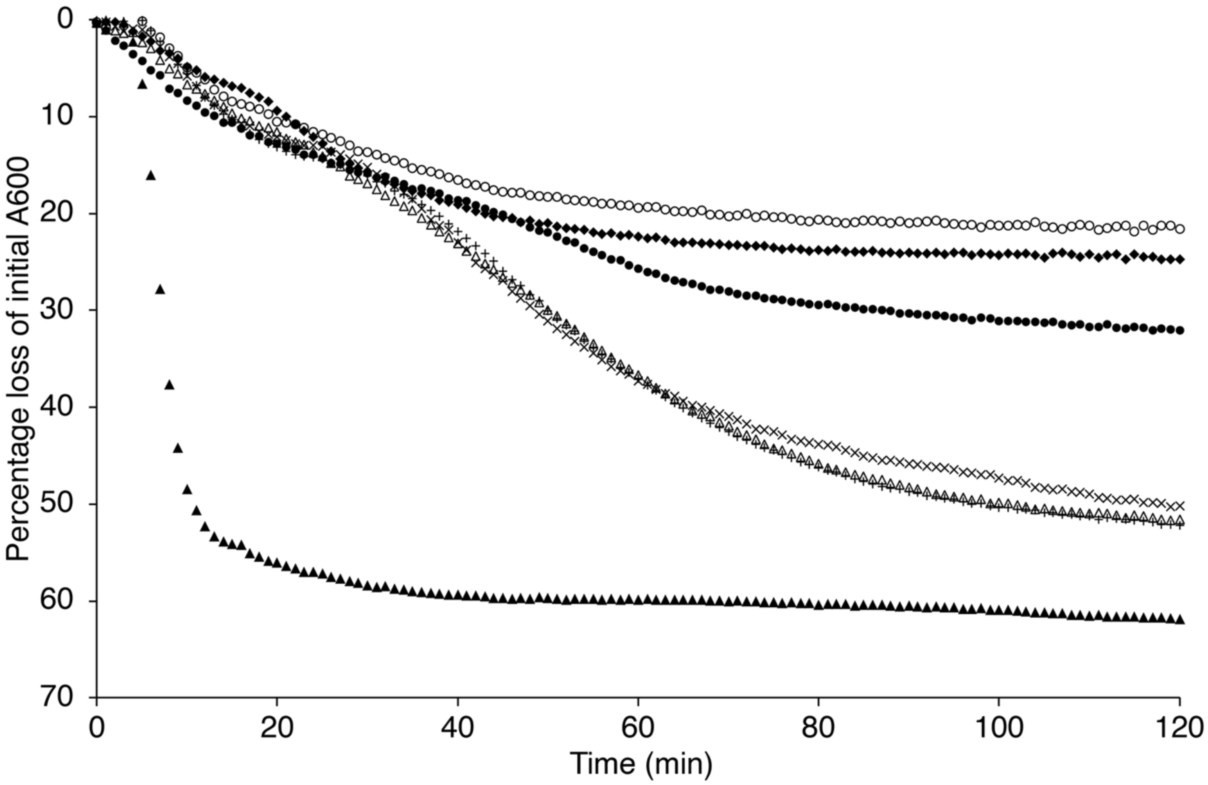

Figure S2 Germination of *Bacillus cereus* spores with variant SleB^{N} proteins. *B. cereus CLE3* mutant spores complemented with plasmid borne variant *sleB* genes were heat shocked at 75°C for 30 minutes and then cooled on ice before resuspending to an absorbance (600 nm) of 1.0 in buffer (10 mM Tris-HCl, pH 7.8) supplemented with 10 mM L-alanine plus 1 mM inosine. Absorbance measurements at 600 nm were recorded at 1 minute intervals as described in the Materials and Methods. Presented data are from single experiments, which are representative of analyses conducted with at least two independent batches of spores and where SD from mean values is <10%. Key: wild type, filled triangles; CLE3 mutant, filled diamonds; native SleB, +; SleB D65A, ×; SleB D88A, open triangles; SleB D65A D88A, closed circles; pHT plasmid control, open circles.

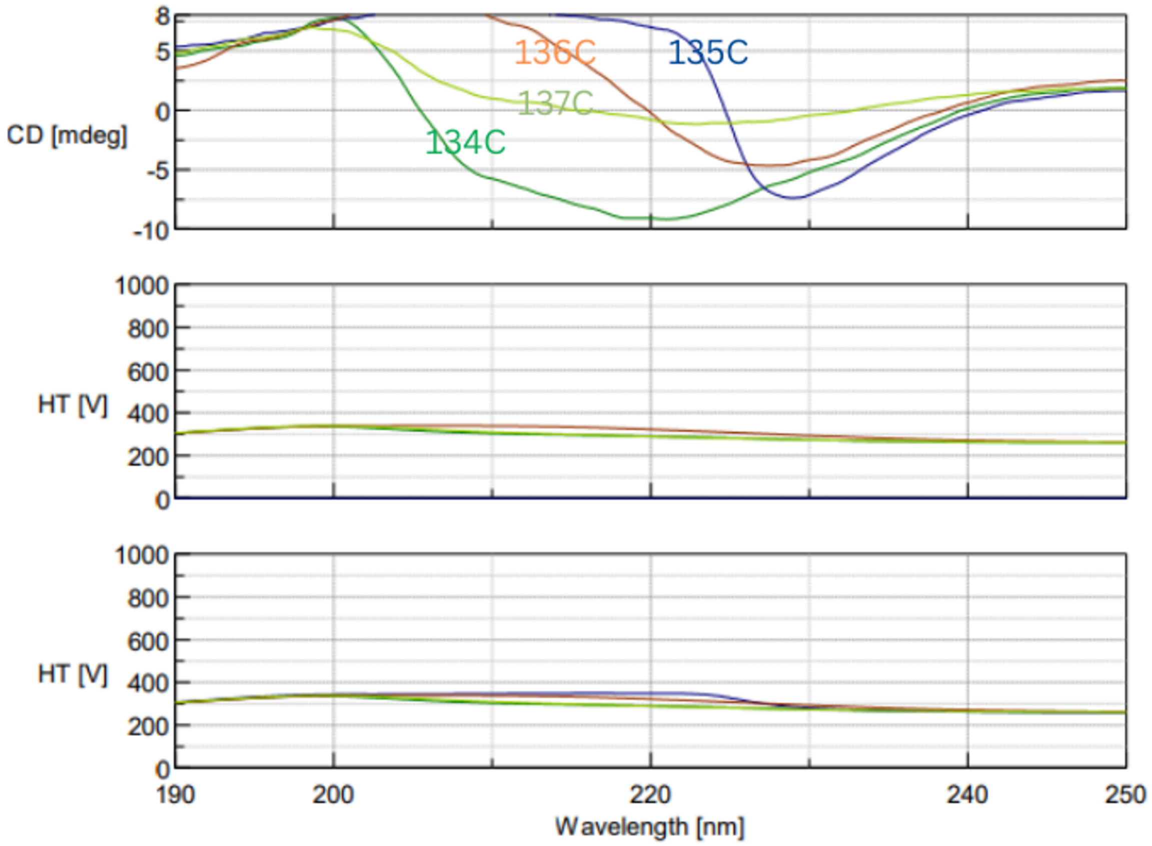

Figure S3 Circular dichroism spectra for recombinant SleB^N proteins. Key: 134C, SleB^N (residues A32-T103 plus N-terminal His_6); 135C, SleB^N-His6 D88A; 136C, SleB^N-His6 D65A; 137C SleB^N-His6 D65A D88A. Proteins were buffer exchanged in 20 mM $NaPO_4$, pH 7.0, and diluted to a final concentration of 2.3 µM. CD spectra were obtained as described in the Materials and Methods.

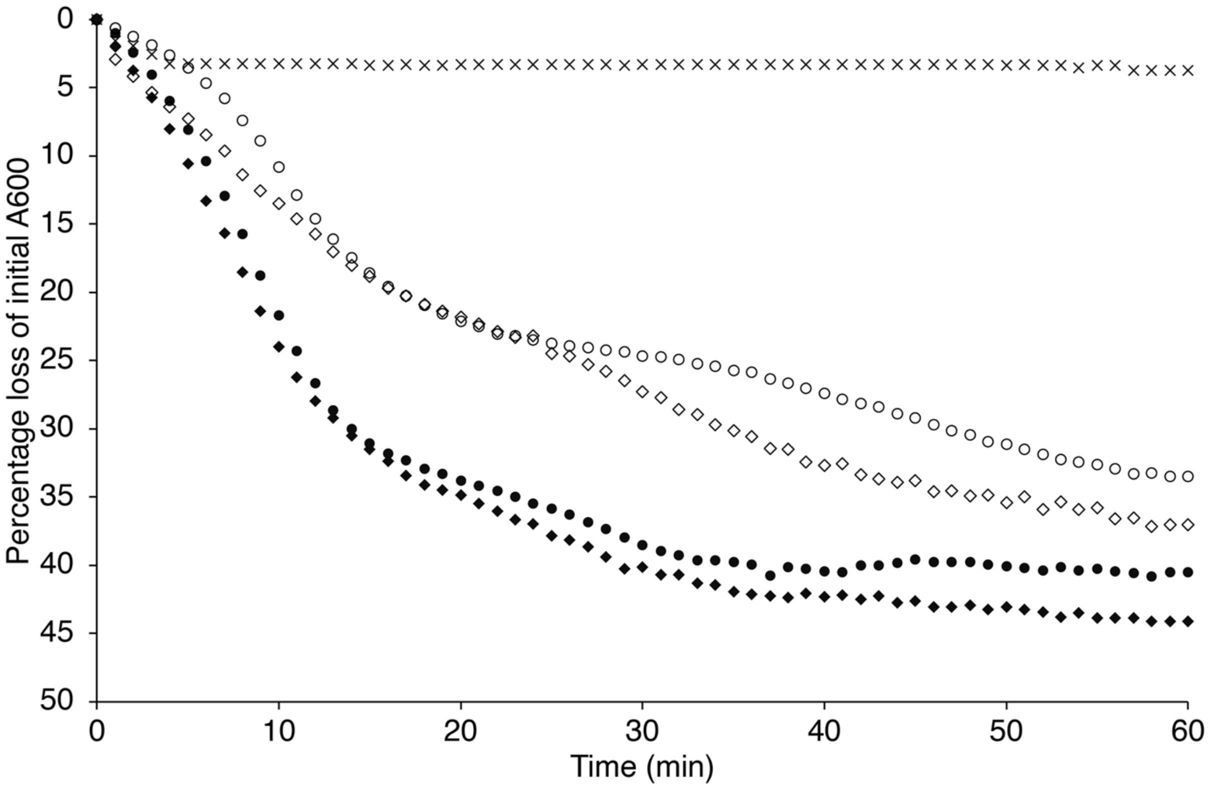

Figure S4 Germination of *B. cereus* spores null for *yhcN* and *ylaJ* in response to dodecylamine. Spores were resuspended to an absorbance (600 nm) of 1.0 in buffer (10 mM Tris-HCl, pH 7.8) supplemented with 1 mM dodecylamine. Samples were incubated at 40°C and A600 values recorded at 1 minute intervals. Presented data are from single experiments, which are representative of analyses conducted with at least two independent batches of spores and where SD from mean values is <10%. Key: wild type spores in buffer without dodecylamine, ×; with dodecylamine: wild type, closed circles; *yhcN ylaJ*, open circles; *cwlJ1 cwlJ2*, closed diamonds; *cwlJ1 cwlJ2 yhcN ylaJ*, open diamonds.

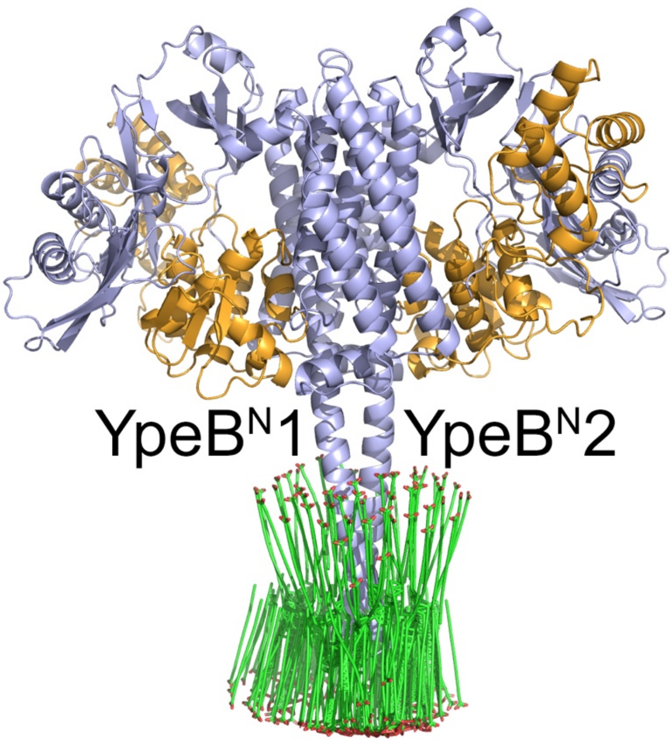

Figure S5 AlphaFold 3 model of the *B. cereus* SleB-YpeB complex embedded in a lipid bilayer. With input queries comprising YpeB and approximately 200 palmitic acid moieties, AlphaFold 3 predicts that the N-terminal region of the former is likely to localise within the assembled lipid bilayer (the choice of palmitic acids and not phospholipids reflecting current AF3 ligand constraints). Key: YpeB, blue-grey; SleB, orange; palmitic acid, green/red.

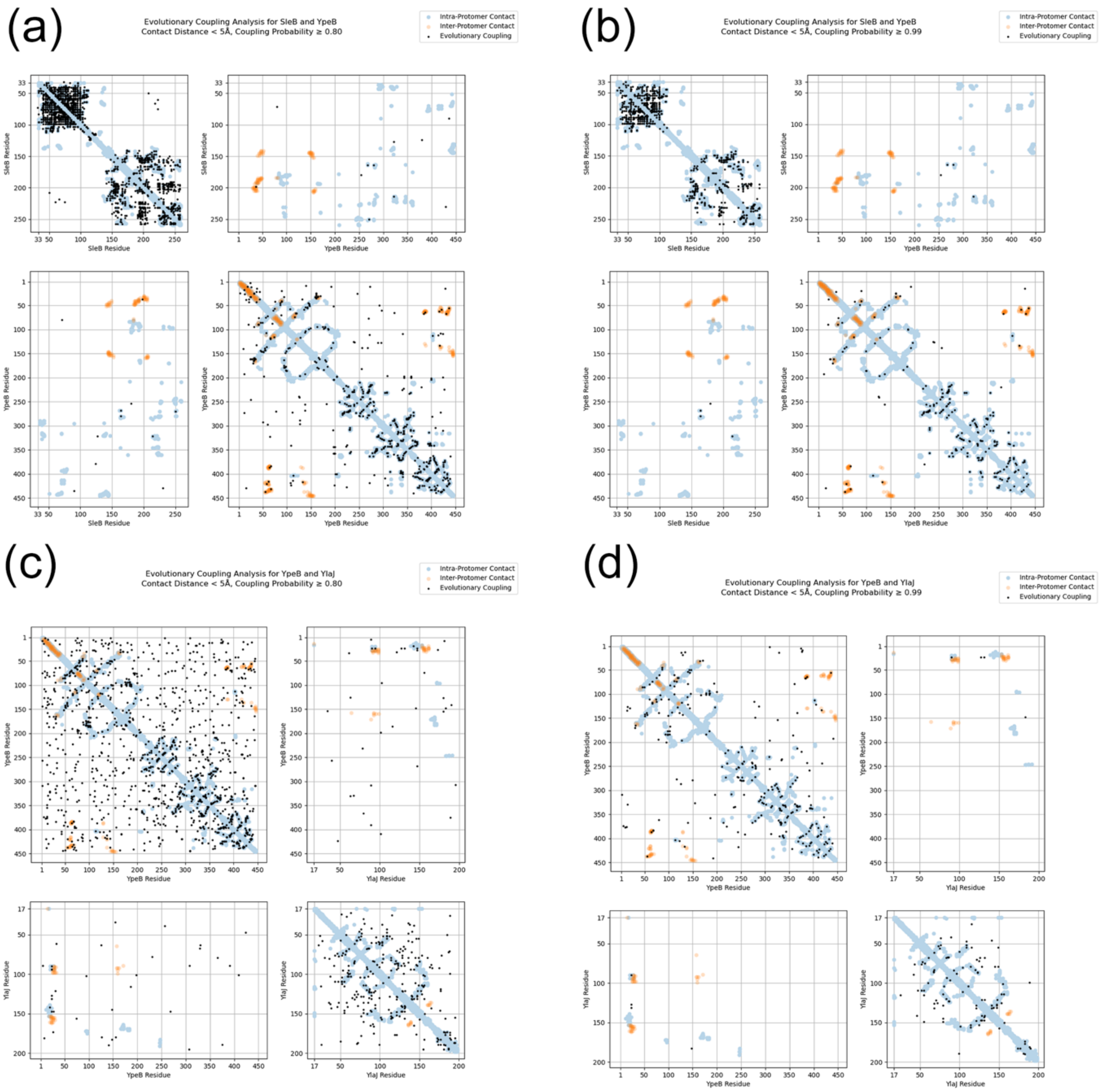

Figure S6 Evolutionary covariance analysis scatter plots for (a) SleB and YpeB, contact distance <5 Å, coupling probability >0.8; (b) SleB and YpeB, contact distance <5 Å, coupling probability >0.99; (c) YpeB and YlaJ, contact distance <5 Å, coupling probability >0.8; (d) YpeB and YlaJ, contact distance <5 Å, coupling probability >0.99. Key: blue dots denote intra-protomer contacts, orange dots inter-protomer contacts, black dots evolutionary coupled residues.
